# A Ruthenium(II) Water Oxidation Catalyst Containing a pH-Responsive Ligand Framework

**DOI:** 10.1021/acs.inorgchem.1c01646

**Published:** 2021-08-10

**Authors:** Fabian L. Huber, Anna M. Wernbacher, Daniel Perleth, Djawed Nauroozi, Leticia González, Sven Rau

**Affiliations:** † Institute of Inorganic Chemistry I, Ulm University, Albert-Einstein-Allee 11, Ulm 89081, Germany; ‡ Institute of Theoretical Chemistry, Faculty of Chemistry, University of Vienna, Währinger Strasse 17, Vienna 1090, Austria

## Abstract

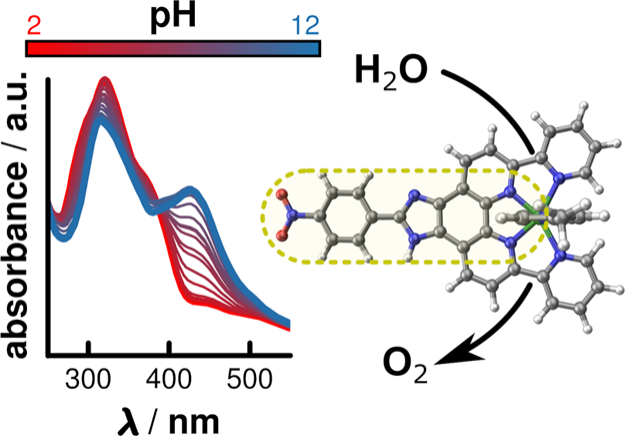

The synthesis of a new Ru^II^-based water oxidation catalyst is presented, in which a nitrophenyl group is introduced into the backbone of dpp via a pH-sensitive imidazole bridge (dpp = 2,9-di-(2′-pyridyl)-1,10-phenanthroline). This modification had a pronounced effect on the photophysical properties and led to the appearance of a significant absorption band around 441 nm in the UV–vis spectrum upon formation of the monoprotonated species under neutral conditions. Theoretical investigations could show that the main contributions to this band arise from transitions involving the imidazole and nitrophenyl motif, allowing us to determine the p*K*_a_ value (6.8 ± 0.1) of the corresponding, twofold protonated conjugated acid. In contrast, the influence of the nitrophenyl group on the electrochemical properties of the catalytic center was negligible. Likewise, the catalytic performance of Ru(dppip-NO_2_) and its parent complex Ru(dpp) was comparable over the entire investigated pH range (dppip-NO_2_ = 2-(4-nitrophenyl)-6,9-di(pyridin-2-yl)-1*H*-imidazo[4,5-*f*][1,10]phenanthroline). This allowed the original catalytic properties to be retained while additionally featuring a functionalized ligand scaffold, which provides further modification opportunities as well as the ability to report the pH of the catalytic solution via UV–vis spectroscopy.

## Abstract 2

Modification of water oxidation catalysts results in a pH-sensitive ligand scaffold. Detailed UV−vis spectroscopic and theoretical studies show that changes in the pH have a significant impact on the photophysical behavior of the compound. Despite this, electrochemical investigations suggest no significant alteration of the redox properties of the catalytic center and photocatalytic experiments reveal that the catalytic performance of the original complex could be retained.

## Introduction

Today, the demand for energy is mainly met by fossil fuels, which has a profound impact on climate change. An attractive option to address these challenges is the use of solar radiation as a sustainable energy source, especially since the amount of sunlight that strikes the earth within only 1 h (4.3 × 10^20^ J) is greater than the earth’s energy consumption in a year (4.1 × 10^20^ J in 2001).^1^ By converting solar energy into chemical energy via artificial photosynthesis, the energy is stored in the form of chemical bonds and can be released when needed.^2,3^ A common example is the production of hydrogen as an energy carrier by splitting water into O_2_ and H_2_ (eq 1).
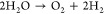
1

This process can be subdivided into two half reactions. In the first half reaction, water is oxidized to dioxygen affording protons and electrons (eq 2). On the other hand, the second half reaction corresponds to the formation of two equivalents of dihydrogen by combining the protons and electrons produced in the first half reaction (eq 3).
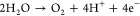
2
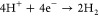
3

Due to the high activation barrier of four proton-coupled electron transfers (PCETs) and the endothermicity of the oxygen–oxygen bond formation, the oxidation of water is considered to be the bottleneck of water splitting.^4−7^ Metal complexes, especially systems based on ruthenium, have attracted considerable interest as active catalysts for water oxidation.^8−10^ In addition, such molecular water oxidation catalysts (WOCs) can be modified via the introduction of different functional groups into the ligand framework. Such modifications, however, usually have a strong influence on the redox properties of the catalyst, which in some cases might not be desirable.

**Figure 1:**
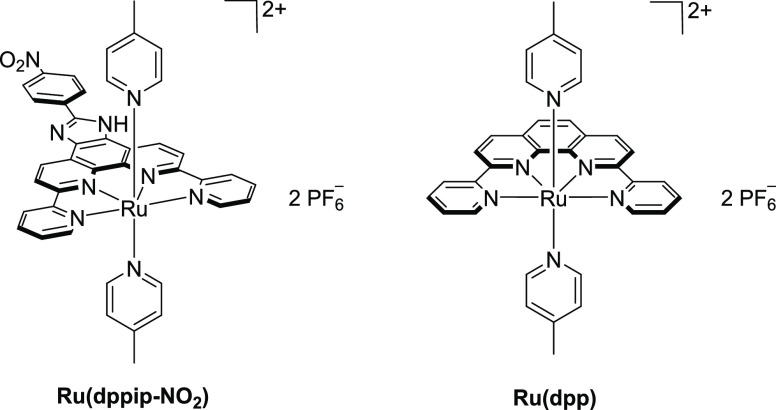
Structure of Ru(dppip-NO_2_) and Ru(dpp).

One established example is [Ru(dpp)(pic)_2_](PF_6_)_2_ (Ru(dpp); dpp = 2,9-di-(2′-pyridyl)-1,10-phenanthroline; pic = 4-picoline), originally published by Thummel *et al.*(11,12) The redox properties of this complex were especially susceptible to modifications of the axial ligands. However, converting the dpp backbone to a phenazine derivative (dppdppz = 3,6-di-(pyrid-2′-yl)-dipyrido[3,2-*a*:2′,3′-*c*]phenazine) had only a minor impact on the catalytic performance.^13^ We were therefore interested whether it would be possible to introduce alternative functional groups at the dpp backbone, which could serve as a starting point for further modifications. Furthermore, we wanted to explore if such modifications retained the innocent character with respect to the catalytic performance. As functionalization of highly substituted phenanthrolines requires a facile access, we utilized the literature-known quinone moiety of dpp for the formation of an imidazolyl moiety. To this end, a nitrophenyl group was introduced via a pH-sensitive imidazole bridge, thereby giving access to [Ru(dppip-NO_2_)(pic)_2_](PF_6_)_2_ (Ru(dppip-NO_2_), dppip-NO_2_ = 2-(4-nitrophenyl)-6,9-di(pyridin-2-yl)-1*H*-imidazo[4,5-*f*][1,10]phenanthroline (Figure 1).

In the present report, we describe the synthesis of Ru(dppip-NO_2_) and its photophysical and preliminary electrochemical characterization, along with theoretical studies and first photocatalytical water oxidation experiments. We address the questions of how the photophysical properties of the new Ru complex are affected by (i) the chemical modification of the dpp-based acceptor ligand and (ii) the protonation state of the imidazole moiety of dppip-NO_2_. Because the interpretation of the experimental data proved to be difficult, quantum chemical methods were used to complement the experimental spectroscopic studies and to provide insight into the nature of the electronic excitations. Furthermore, pH-dependent light-driven catalytic runs were performed to investigate the influence of the degree of protonation of the ligand scaffold, i.e., of the amphoteric imidazole moiety, on the catalytic behavior of the WOC.

## Results and Discussion

### Synthesis and Structural Characterization

**Scheme 1:**
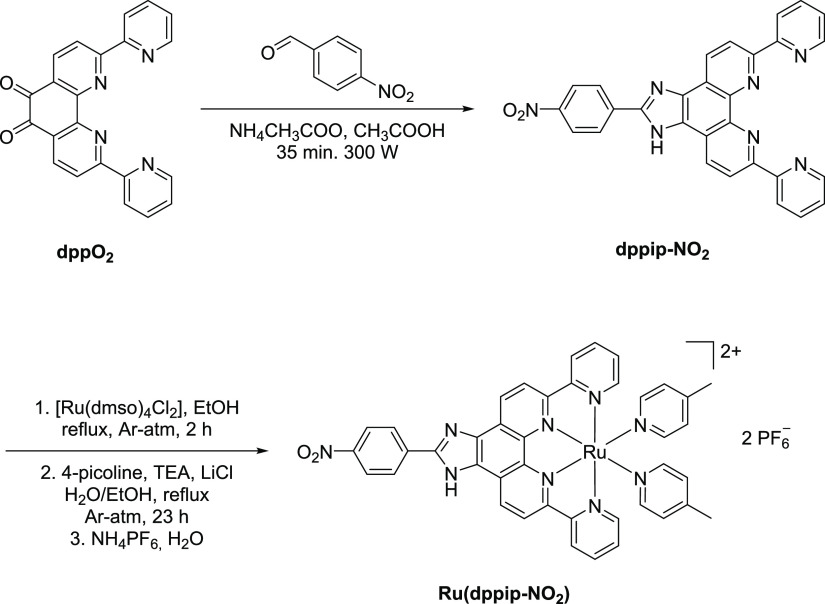
Synthesis Route toward Ru(dppip-NO_2_)

The free ligand dppip-NO_2_ was obtained via a microwave-assisted Debus–Radziszewski reaction of dppO_2_ with 4-nitrobenzaldehyde and ammonium acetate in glacial acetic acid following literature-known procedures (Scheme 1).^14,15^ To prevent solvation of imidazolium cations, it was crucial that the reaction mixture was neutralized with diluted ammonia (25%). This reaction resulted in a good yield of 85%, and the ligand was characterized via ^1^H NMR spectroscopy (Figure S3), while ^13^C NMR measurements were not possible due to low solubility. The constitution of dppip-NO_2_ was further confirmed by high-resolution mass spectrometry (HRMS; Figure S7).

The synthesis of the complex Ru(dppip-NO_2_) followed a modified literature procedure.^12^ Purification of the complex was obtained by washing with water and diethyl ether followed by diffusion crystallization (DMF/Et_2_O), resulting in a moderate yield of 29% of pure Ru(dppip-NO_2_).

The formation of the desired product could be confirmed by ^1^H NMR (Figure S5) and HRMS (Figures S8 and S9). Analysis of H,H-COSY experiments further allowed the assignment of proton signals (Figure S6). Due to the amphoteric behavior of the imidazole unit,^16^ the corresponding proton was not detected via NMR spectroscopy, resulting in a pseudo C_2v_-symmetry for both the complex Ru(dppip-NO_2_) and the free ligand dppip-NO_2_. Characterization via ^13^C NMR spectroscopy was not possible because of a relatively poor solubility of the complex.

Since no experimental crystal structure of Ru(dppip-NO_2_) could be produced so far, theoretical methods were used to gain insight into its structure. The suitability of the computational approach to describe such Ru complexes is justified by the good agreement that was obtained between the computed structure of Ru(dpp) and its experimental crystal structure.^12,13^ We therefore employed the same methodology to investigate Ru(dppip-NO_2_), which was found to exhibit a similar structure like Ru(dpp). In particular, the coordination sphere of the Ru center is hardly affected by the modified equatorial dppip-NO_2_ ligand. The dpp or dppip-NO_2_ ligand binds with four N atoms to Ru exhibiting two short Ru–N bonds to the central phenanthroline ring (1.96 Å) and two elongated Ru–N bonds of about 2.18–2.19 Å to the peripheral pyridine substituents of the equatorial ligand. The rather large N–Ru–N bond angle of these peripheral pyridines of 125–126° (compared to 90° in an ideal octahedral geometry) and long Ru–N bond lengths were suggested to facilitate an attack of water in the case of Ru(dpp).^13,17^

**Figure 2:**
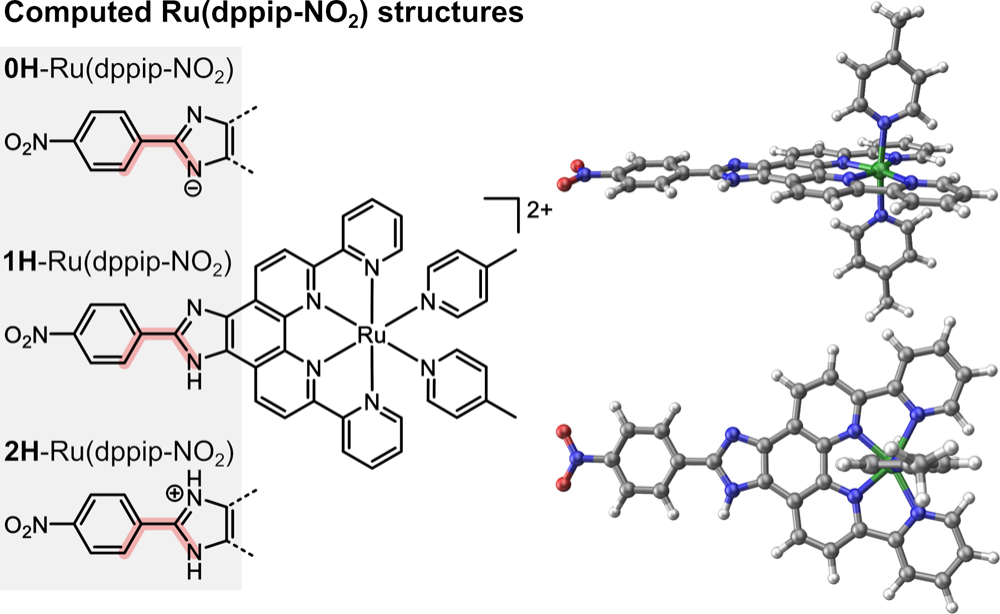
Computed Ru(dppip-NO_2_) structures with the deprotonated imidazole moiety (0H-Ru(dppip-NO_2_)), with one proton (1H-Ru(dppip-NO_2_)), and with two protons on the imidazole (im) group (2H-Ru(dppip-NO_2_)). The nitrophenyl dihedral angle with respect to the im-dpp ring system is highlighted in red. The geometry of 1H-Ru(dppip-NO_2_) is shown on the right (side view and top view).

**Figure 3:**
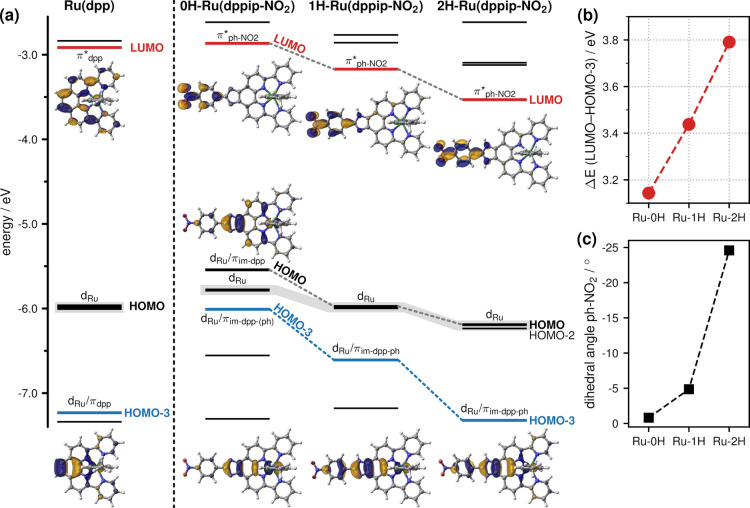
(a) Calculated energetic order of frontier molecular orbitals (MOs) of 0H,1H,2H-Ru(dppip-NO_2_); d_Ru_ orbitals are shaded in gray, and the HOMO-3 and LUMO are highlighted in blue and red, respectively. (b) LUMO–HOMO-3 gap. (c) Dihedral angles of the nitrophenyl group with respect to the im-dpp ring system.

Due to the featured imidazole motif of Ru(dppip-NO_2_), the structure of the complex depends on the degree of protonation of the imidazole moiety. To investigate this dependency, structures with a double-protonated (2H-Ru(dppip-NO_2_)) and deprotonated imidazole group (0H-Ru(dppip-NO_2_)) were calculated in addition to the parent complex 1H-Ru(dppip-NO_2_), as indicated in Figure 2. Interestingly, the nitrophenyl group of dppip-NO_2_ is slightly rotated with respect to the imidazole-dpp ring system, depending on the degree of protonation. Specifically, the nitrophenyl dihedral angle, highlighted in red in Figure 2, increases with the number of protons/H-atoms on the imidazole group in the following order, probably due to steric effects: 0H-Ru(dppip-NO_2_) (nearly planar, −0.8°) < 1H-Ru(dppip-NO_2_) (−5°) < 2H-Ru(dppip-NO_2_) (−25°) (cf. Figure 3c, see also Figure S10 and Table S1).

### Photophysical Properties

To characterize the new Ru(dppip-NO_2_) complex and to better understand its difference from Ru(dpp), UV–vis spectra of both complexes were recorded. It is very surprising to note that the new complex Ru(dppip-NO_2_) shows a very intense orange-red color once dissolved in acetonitrile. The recorded UV–vis spectra show a maximum at 441 nm. This feature is completely absent in the parent compound Ru(dpp) under identical conditions. Addition of base and acid to these probes shows that, for Ru(dppip-NO_2_), absorption bands change in intensity and energy, whereas no such behavior could be observed for Ru(dpp). As this interesting and unexpected behavior could not be understood from a purely experimental perspective, more detailed investigations have been performed. Thus, in the following, the results of the photophysical and theoretical investigations will be described together.

### Electronic Structure

Figure 3a shows the computed energetic order of the frontier molecular orbitals (MOs) of 1H-Ru(dppip-NO_2_) and its (de)protonated forms compared to Ru(dpp). The gap between the highest occupied MO (HOMO) and lowest unoccupied MO (LUMO) decreases from 3.1 eV in Ru(dpp) to 2.8 eV in 1H-Ru(dppip-NO_2_) and ca. 2.7 eV in the (de)protonated complexes. In addition, the HOMO-3 and LUMO of Ru(dpp) and of 0H,1H,2H-Ru(dppip-NO_2_) are depicted as they are involved in the electronic excited states underlying the UV–vis absorption spectra (see below in the next section). While the HOMO–LUMO gap in 0H,1H,2H-Ru(dppip-NO_2_) remains similar, the energy of the HOMO-3 decreases with the number of protons on dppip-NO_2_, thus increasing the LUMO–HOMO-3 gap (Figure 3b). Other relevant highest occupied and lowest unoccupied MOs can be found in Figure S11.

In both Ru(dpp) and (1H),2H-Ru(dppip-NO_2_), the first three d_Ru_-based HOMOs, which are shaded in gray in Figure 3a, are nearly degenerate. The HOMO of 1H-Ru(dppip-NO_2_) also has a contribution from the dppi ligand, but the d_Ru_ character is still dominant. In contrast, the planar 0H-Ru(dppip-NO_2_) complex exhibits a HOMO of d_Ru_/π_im-dpp_ character, which is slightly higher in energy than the d_Ru_-based HOMO-1,2. The d_Ru_/π_im-dpp-(ph)_ HOMO-3 already exhibits a contribution of the imidazole group and to a lesser extent of the phenyl ring (ph) in 1H-Ru(dppip-NO_2_) as well as in its (de)protonated forms.

A major difference between Ru(dpp) and Ru(dppip-NO_2_) becomes apparent in the LUMO, which is centered on the nitrophenyl group in the latter complex (π*_nitrophenyl_). The subsequent higher-lying unoccupied MOs as well as the LUMO in Ru(dpp) are of π*_dpp_ character in all complexes. The LUMO+3 in Ru(dpp), LUMO+4 in 0H,1H-Ru(dppip-NO_2_), and LUMO+5 in 2H-Ru(dppip-NO_2_) are of π*_pic_ character.

**Figure 4:**
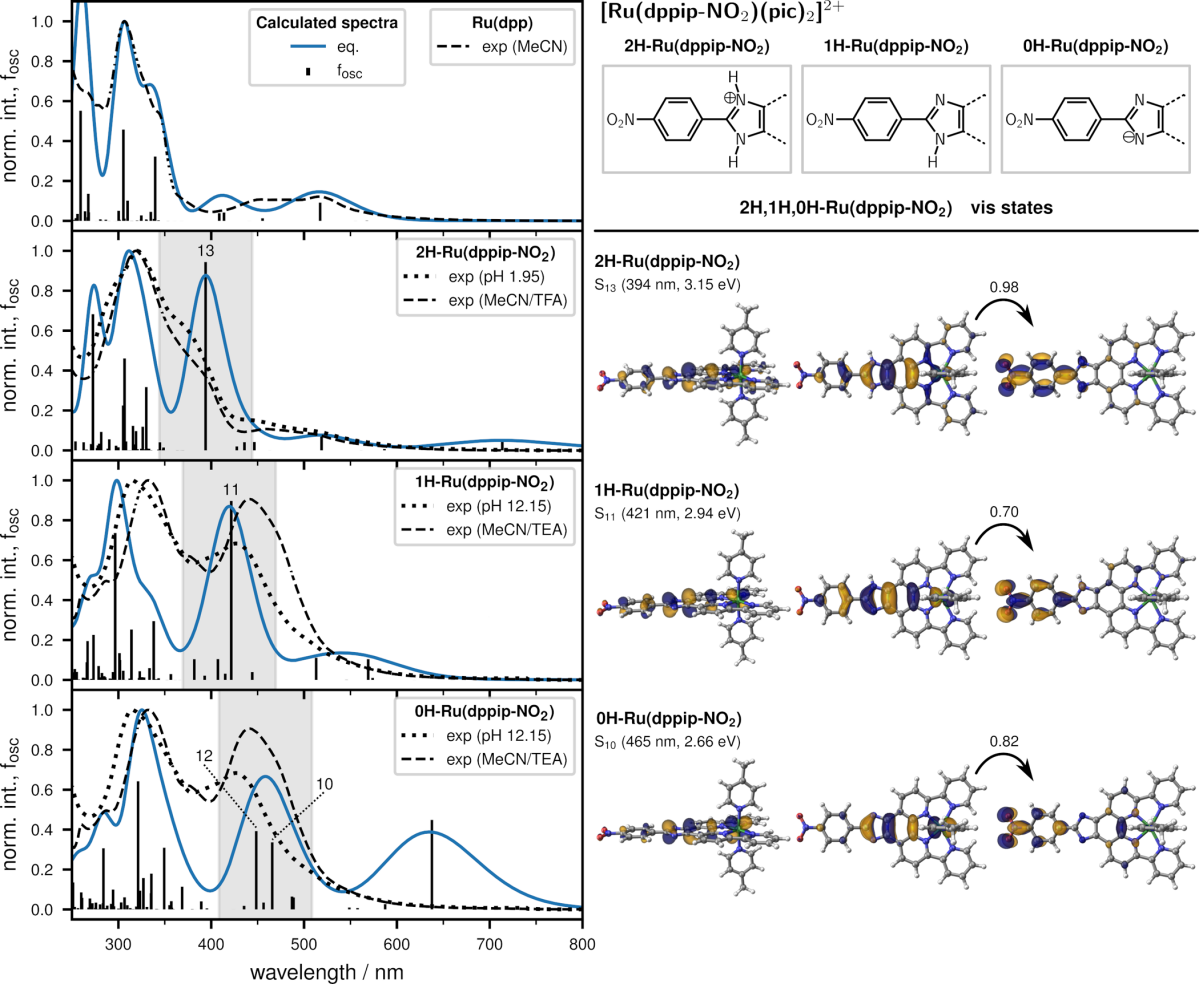
Calculated UV–vis absorption spectra of Ru(dpp) and 2H,1H,0H-Ru(dppip-NO_2_) equilibrium geometries in MeCN, and oscillator strengths *f*_osc_ compared to the experimental spectra of Ru(dpp) in MeCN and Ru(dppip-NO_2_) in MeCN/TFA, MeCN/TEA (dashed line), and Britton–Robinson buffer/MeCN (dotted line). For the calculated spectra of the equilibrium geometries, Gaussian functions with a full width at half-maximum fwhm of 0.35 eV were used. No shifting has been applied. The natural transition orbitals of the intense vis states of 2H,1H,0H-Ru(dppip-NO_2_) are shown on the right, and the structures with two, one, or zero protons on the imidazole group of the dppip-NO_2_ ligand are indicated at the top. (B3LYP-D3BJ, ZORA, ZORA-def2-TZVP, and ZORA-TZVP on Ru, C-PCM (MeCN)).

Based on these reference points, detailed investigations were performed. Figure 4 compares the experimental UV–vis spectra of Ru(dpp) recorded in acetonitrile (MeCN), Ru(dppip-NO_2_) in MeCN/triethylamine (TEA, 0H,1H-Ru(dppip-NO_2_)), and MeCN/trifluoroacetic acid (TFA, protonated 2H-Ru(dppip-NO_2_)) with the computed spectra in MeCN (convoluted spectra based on the corresponding equilibrium geometries in blue). In addition, a UV–vis titration of Ru(dppip-NO_2_) in 80 vol-% Britton–Robinson buffer and 20 vol-% MeCN was performed (Figure 4) and the computed spectra of the protonated and deprotonated complexes are compared to the experimental spectra of the titration at pH values of 1.95 and of 12.15, respectively.

The excited states were first characterized by inspection of the natural transition orbitals (NTOs). NTO pairs of states contributing to the strong absorption band in the visible region in 2H,1H,0H-Ru(dppip-NO_2_) are shown in Figure 4 on the right. Furthermore, an automatized analysis of the transition density matrix was performed with TheoDORE,^18−20^ enabling a classification of the states in terms of their charge transfer character.^21^ The states can be labeled as locally excited ligand-centered (LC) states or charge transfer excitations, e.g., metal-to-ligand charge transfer (MLCT) or ligand-to-ligand charge transfer (LLCT). Ligand-to-metal charge transfer (LMCT) excitations and metal centered Ru d–d excitations were not found to be important for the absorption spectra of these Ru complexes.

The most prominent difference in the spectra of Ru(dpp) and Ru(dppip-NO_2_) is the strong absorption band of the latter in the visible energy range, which is not observed in Ru(dpp). Here, 1H-Ru(dppip-NO_2_) features a state of high intensity, the S_11_ at 421 nm (2.9 eV), which is due to an excitation to the nitrophenyl group, as can be seen in the NTO pairs shown in Figure 4 on the right. S_11_ mostly corresponds to a d_Ru_/π_im-dpp-ph_ → π*_nitrophenyl_ LLCT/MLCT excitation with a contribution of a d_Ru_ → π*_dpp,NO2_ MLCT excitation. As shown in Figure S13, a decomposed spectrum of 1H-Ru(dppip-NO_2_) taking only states without a significant contribution of the nitrophenyl group into account closely resembles the spectrum of Ru(dpp). The differences from Ru(dpp) in the visible energy range seem to be a consequence of transitions to the nitrophenyl-centered LUMO in 1H-Ru(dppip-NO_2_).

Moreover, the absorption band of Ru(dppip-NO_2_) in the visible energy range, which is highlighted by the gray bars in Figure 4, is pH-dependent. The calculated band maximum shifts to higher energies with increasing number of protons in the order of 2H-Ru(dppip-NO_2_) 394 nm < 1H-Ru(dppip-NO_2_) 419 nm < 0H-Ru(dppip-NO_2_) 458 nm, while the character of the involved states stays similar (cf. NTOs in Figure 4 and Table S2). In all complexes, they can be described by a d_Ru_/π_im-dpp-ph_ → π*_nitrophenyl_ excitation (mostly HOMO-3 → LUMO), where the d_Ru_ contribution decreases and the contribution of the phenyl ring increases with increasing number of protons. Both 1H-Ru(dppip-NO_2_) and 0H-Ru(dppip-NO_2_) exhibit an additional Ru/(dppi) → dpp MLCT contribution, which increases the overall MLCT character of the absorption band. In 0H-Ru(dppip-NO_2_), another intense state (S_12_) contributes to the band in the visible region, which is predominantly of d_Ru_ → π*_dpp_ MLCT character. The shift of vis states S_10_, S_11_, and S_13_ of 0H,1H,2H-Ru(dppip-NO_2_) to higher energies correlates with an increase in the LUMO–HOMO-3 gap (see Figure 3b). Considering that these states consist of predominantly HOMO-3 → LUMO character in all complexes, this correlation suggests that the stabilization of the HOMO-3 with increasingly positive charge on the imidazole group contributes to the increase in excitation energy in the order of 0H-Ru(dppip-NO_2_) < 1H-Ru(dppip-NO_2_) < 2H-Ru(dppip-NO_2_). A similar blue shift of the vis band upon protonation was reported for a Ru complex bearing a 2,2′-biimidazole ligand, which was also attributed to a stabilization of d_Ru_/π orbitals that contribute to the electronic excitation.^22^

Like Ru(dpp), Ru(dppip-NO_2_) is not emissive in MeCN and CH_2_Cl_2_ at room temperature.^12,13^ A likely explanation for the absence of any appreciable emission is the highly distorted coordination geometry between the tetradentate dpp-sphere and the ruthenium center, which lowers the d–d states and allows for non-radiative relaxation of the excited state.^12,13^ Similar conclusions have been drawn for the negligible emission at room temperature of [Ru(tpy)_2_]^2+^.^23,24^

### p*K*_a_ Determination

As briefly discussed above, the pH value of the surrounding solution significantly influences the photophysical properties of Ru(dppip-NO_2_). During the course of titration experiments, a rising band at 425.5 nm and a decreasing intensity for the band at 320.5 nm upon increasing the pH were observed, along with the formation of an isosbestic point at 384 nm. These spectral changes in absorption bands at 320.5 and 425.5 nm could be used to determine the p*K*_a_ value of Ru(dppip-NO_2_) by using a sigmoidal fit on the titration curves, which yield inflection points at p*K*_a_^320.5^ = 7.06 ± 0.07 and p*K*_a_^425.5^ = 6.59 ± 0.05 (Figure S14).^22,25,26^ Based on these two values, a p*K*_a_ of 6.8 ± 0.1 was determined, which likely corresponds to the twofold protonated form of the imidazole motif (2H-Ru(dppip-NO_2_)), as other imidazoles have similar p*K*_a_ values for their respective conjugated acids.^27^ Likewise, the p*K*_a_ corresponding to the mono-protonated imidazole motif was expected to be around 12–14,^27^ and the exact value however could not be determined as the addition of additional NaOH resulted in phase separation between MeCN and the aqueous buffer solution as well as partial precipitation of Ru(dppip-NO_2_).

**Figure 5:**
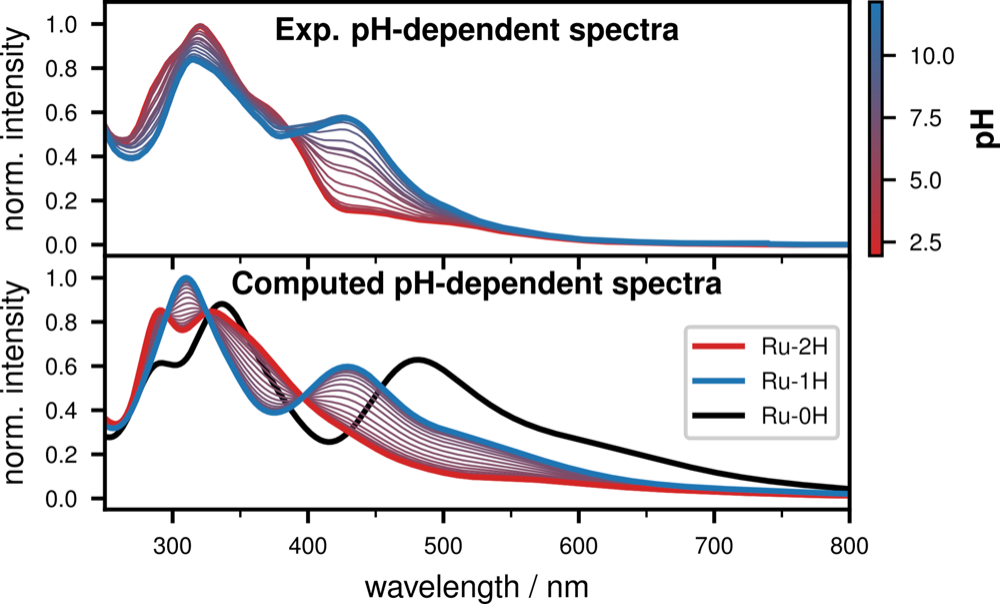
Experimental pH-dependent UV–vis spectra of Ru(dppip-NO_2_) in Britton–Robinson buffer/MeCN (top) and computed pH-dependent spectra (bottom). For the latter, the Wigner spectra of 2H,1H-Ru(dppip-NO_2_) were used and their intensities scaled based on an experimental p*K*_a_ of 6.8. In addition, the Wigner spectrum calculated for 0H-Ru(dppip-NO_2_) is shown in black. (B3LYP-D3BJ, ZORA, ZORA-def2-TZVP, and ZORA-TZVP on Ru, C-PCM (MeCN)).

A dependence of the vis absorption band on the protonation state of the imidazole group is also shown by computing pH-dependent spectra using the spectra of 2H-Ru(dppip-NO_2_) and 1H-Ru(dppip-NO_2_) and scaling their intensities based on an experimental p*K*_a_ of 6.8. To account for vibrational effects, the spectra of 2H,1H,0H-Ru(dppip-NO_2_) were calculated using 50 geometries for each Ru complex sampled from a temperature-dependent Wigner distribution at 300 K. Figure 5 compares the computed pH-dependent spectra with the experimental spectra of Ru(dppip-NO_2_) in Britton–Robinson buffer/MeCN.

A good agreement is obtained between the spectra calculated in this way and the pH-dependent experiments. The increase in intensity in the visible energy range with increasing pH is clearly shown, which is due to the dppip-NO_2_-dominated band of 1H-Ru(dppip-NO_2_) at 429 nm and lower energies in the calculated spectrum. The absorption band maximum of the protonated complex 2H-Ru(dppip-NO_2_), on the other hand, is only observed in the UV range at 329 nm. In contrast, the B3LYP spectra suggest that the deprotonated complex 0H-Ru(dppip-NO_2_) shows an intense band at rather low energies (481 nm, black spectrum). This might indicate that it does not contribute significantly to the experimental pH-dependent spectra in the investigated pH range. The deprotonated complex was hence not considered in the computed pH-dependent spectra. The changes in the absorption band are mainly caused by the shift of the excitation energy of the intense states in the visible energy range to higher energies with increasing number of protons on the dppip-NO_2_ ligand.

### Electrochemistry

The electrochemical features of Ru(dppip-NO_2_) were investigated by cyclic voltammetry in 0.1 M solution of *n*Bu_4_NPF_6_ in DMF using a glassy carbon working electrode, a silver reference electrode, and a Pt-wire as the counter electrode. Potentials were referenced against ferrocene/ferricenium (Fc/Fc^+^) (Figure S15). The pertinent data is summarized in Table 1.

**Table 1:** Electrochemical Potentials *E* vs Fc/Fc^+^ [V] for Ru(dppip-NO_2_), dppip-NO_2_, and Ru(dpp)

	*E*_ox_	*E*_red1_	*E*_red2_	*E*_red3_	*E*_red4_
Ru(dppip-NO_2_)	0.64	–1.07	–1.24	–1.49	–1.79
dppip-NO_2_		–1.03	–1.35	–1.65	
Ru(dpp)	0.67	–1.42	–1.65		

In the anodic region, Ru(dppip-NO_2_) showed a quasi-reversible oxidation at 0.64 V, which can likely be assigned to the first oxidation of the ruthenium center (Ru(II/III)). In the cathodic region, the complex featured multiple irreversible reductions around −1.07, −1.24, −1.49, and −1.79 V. These redox events presumably are ligand-based as suggested by a comparison with the electrochemical features of dppip-NO_2_ showing irreversible reduction processes at −1.03, −1.35, and −1.65 V (Figure S16). Interestingly, the introduction of the imidazole moiety at the periphery of the phenanthroline seems not to impact the redox properties of the catalytic center significantly as only a slight cathodic shift of −0.03 V is observed for the oxidation of Ru(II/III) in Ru(dpp). It is worth noting that Ru(dppip-NO_2_) suffers degradation upon electrochemical cycling as observed in new features between −0.7, 0.3, and 0.52 V (Figure S16).

### Photocatalytical Water Oxidation

For light-driven water oxidation, a three-component system was investigated consisting of the catalyst Ru(dppip-NO_2_) (2.6 μM), the photosensitizer [Ru(dceb)_2_(bpy)](PF_6_)_2_ (0.3 M, PS, dceb = diethyl[2,2′-bipyridine]-4,4′-dicarboxylate), and Na_2_S_2_O_8_ (10 mM) as the sacrificial agent in aqueous H_3_BO_3_/NaHCO_3_ buffer (5 mL, 0.08 M H_3_BO_3_ containing 0.2 mL of MeCN to improve solubility). The catalytic runs were carried out in sealed glass vessels equipped with two oxygen sensor spots, which allowed for independent measurement of the oxygen concentration in the gas and the liquid phase;^28^ further, the samples were stirred constantly during the whole run.

**Figure 6:**
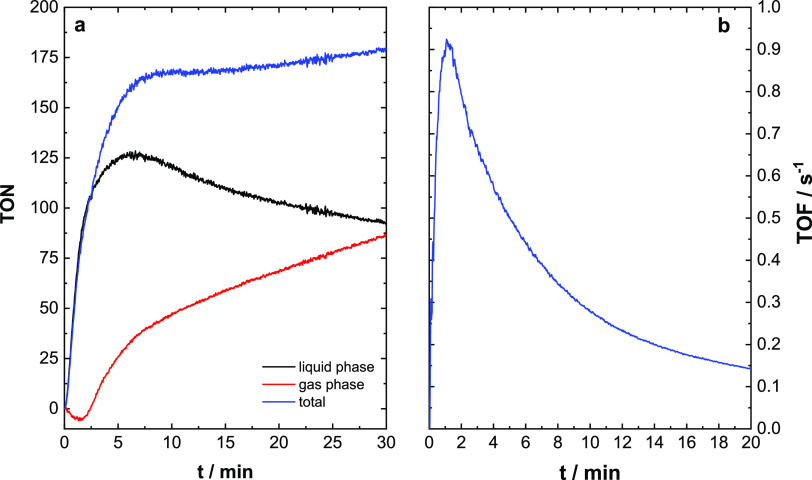
Representative catalytic run of Ru(dppip-NO_2_) at pH 6.07. (a) TON as determined in the respective phases (liquid and gas) and the total TON (liquid + gas phase). (b) TOF (turnover frequency) development over the catalytic run based on the total TON. Conditions: Ru(dppip-NO_2_), 2.6 μM; PS, 0.3 mM; and Na_2_S_2_O_8_, 10 mM; solvent: 96 vol-% aqueous H_3_BO_3_/NaHCO_3_ buffer (0.08 M H_3_BO_3_) and 4 vol-% MeCN. Irradiation with one LED stick, λ_max_ = 470 nm, ca. 50 mW cm^–2^, continuous stirring during the reaction.

Figure 6a depicts a catalytic run of Ru(dppip-NO_2_) conducted at pH 6.07, where the oxygen concentrations of the individual phases (liquid and gas) are measured and the total TON (turnover number) is calculated from those measurements.

As shown in Figure 6a, the overall O_2_ concentration rose rapidly upon irradiation and reached a plateau after *ca.* 8 min, reaching a total TON (liquid + gas phase) of *ca.* 168. In the liquid phase, O_2_ concentration reached its maximum after *ca.* 6 min, after which the O_2_ concentration decreases steadily. The reason behind this is that dissolved O_2_ slowly diffuses from the liquid into the gas phase. After 6 min, this diffusion proceeds faster than the generation of new O_2_, indicating that a decrease in catalytic activity below 0.5 s^–1^ under the utilized conditions results in net loss of O_2_ from the liquid phase.^28^ The overall decrease in catalytic activity becomes more evident by analyzing the development of the TOF (turnover frequency) over the course of the catalytic run (Figure 6b). The maximum TOF of 0.9 s^–1^ was reached in 1 min, corresponding to the initial rapid increase in O_2_, after which the catalytic activity decreases asymptotically, only reaching a TOF of 0.3 s^–1^ after 10 min.

**Figure 7:**
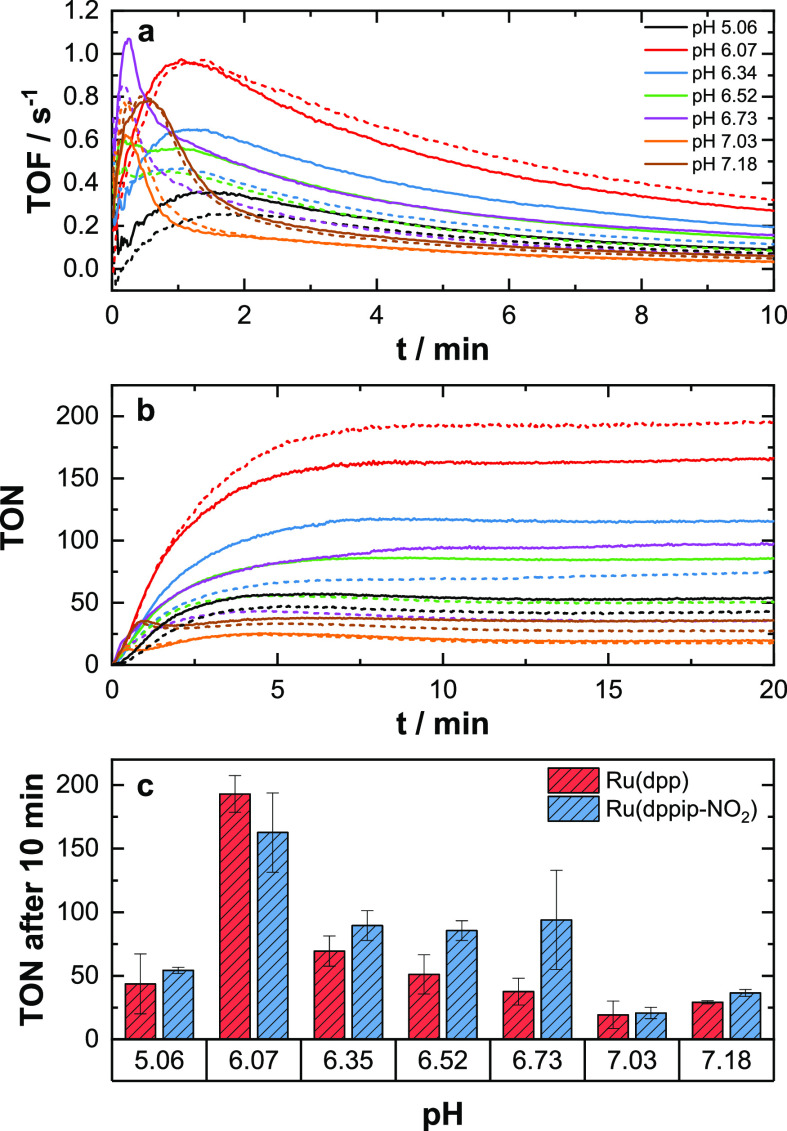
(a) Average total TOFs of the pH screening. (b) Average TONs of the pH screening. Results for Ru(dpp) are given in dotted lines, while solid lines refer to Ru(dppip-NO_2_). (c) Comparison of the overall TONs after 10 min between Ru(dpp) and Ru(dppip-NO_2_). Conditions: Ru(dppip-NO_2_)/Ru(dpp), 2.6 μM; PS 0.3, mM; and Na_2_S_2_O_8_, 10 mM; solvent: 96 vol-% aqueous H_3_BO_3_/NaHCO_3_ buffer (0.08 M H_3_BO_3_) and 4 vol-% MeCN. Irradiation with one LED stick, λ_max_ = 470 nm, ca. 50 mW cm^–2^, continuous stirring during the catalytic run.

However, to determine ideal conditions for the photocatalytic water splitting, water oxidation experiments have been performed at different pH values in a pH screening process. The pH value was adjusted by adding different amounts of solid NaHCO_3_ to the aqueous 0.08 M H_3_BO_3_ solution, thereby creating several borate buffers of different pH values. During this screening, the catalytic activity was determined for each pH value three times and the average TON and TOF were calculated. For reference, the same procedure was applied using Ru(dpp) as the catalyst. Figure 7 summarizes the results of the pH screenings.

Compared to Ru(dpp), Ru(dppip-NO_2_) consistently yields higher amounts of O_2_, with the exception of catalytic runs performed at pH 6.07, where Ru(dpp) reaches a TON of 193 ± 14 compared to 163 ± 31 of Ru(dppip-NO_2_) (Figure 7c). Further, both complexes show the highest catalytic yield at pH 6.07. It is however worth noting that, within the margins of error, both catalysts show very comparable performances. The TOFs of both complexes also behave similarly; it should however be pointed out that Ru(dppip-NO_2_) shows the highest activity at pH 6.73, reaching a TOF of *ca.* 1 s^–1^ (Figure 7a). This activity cannot be maintained for a long time. In contrast, at pH 6.07, a relatively high TOF can be maintained by both catalysts for a longer time, resulting in the highest TONs at pH 6.07.

These findings indicate no significant influence of the nitrophenyl group of the dppip-NO_2_ ligand on the catalytic Ru-center. At pH 6, a 2 e^–^/2 H^+^ process involving a water molecule from [Ru^II^]^2+^ to the seven-coordinated [Ru^IV^(O)]^2+^ was proposed as a possible initial oxidation step during catalysis for Ru(dpp).^29^ Because of the similar activity and structure and the negligible influence of the nitrophenyl group, it is reasonable to assume an analogous mechanism for Ru(dppip-NO_2_).

Nevertheless, it has also been suggested that, prior to active oxygen evolution, an oxygen atom transfer from Ru to the N-atoms of the outer pyridines may take place. This leads to the formation of a di-oxo species as the active catalyst displaying an O,N,N,O tetradentate ligand sphere.^30,31^ To reveal whether, also for Ru(dppip-NO_2_), such a species might represent an intermediate of the catalytic cycle, chemical oxidation of Ru(dppip-NO_2_) using (NH_4_)_2_[Ce(NO_3_)_6_] (CAN) according to a previous literature report was performed.^31^ Mass spectrometry revealed that, by increasing the amount of added CAN, the signal assigned to Ru(dppip-NO_2_) decreased and new peaks that can be assigned to [M-2PF_6_^–^ + O]^2+^ and [M-2PF_6_^–^ + 2O]^2+^ increased in intensity (see Figures S16–S19). Interestingly, by increasing the amount of added CAN from 8 to 12 equiv, peaks that were assigned to [M-2PF_6_^–^ + O]^2+^-derived species vanished and those of the [M-2PF_6_^–^ + 2O]^2+^ species increased. We thus conclude that, also for Ru(dppip-NO_2_), a species containing an O,N,N,O coordination sphere may represent an important intermediate of the catalytic cycle.

## Conclusions

A new dpp-based ligand scaffold and the corresponding WOC, Ru(dppip-NO_2_), could be prepared by introducing a nitrophenyl group to the tetradentate ligand via a pH-sensitive imidazole bridge. This modification had a pronounced effect on the photophysical properties of the complex. Compared to the parent complex Ru(dpp), the new complex exhibits an intense absorption at 441 nm in the singly protonated form. The main contributions to this absorption band are transitions involving the imidazole and nitrophenyl motifs, as shown by TDDFT calculations. Consequently, due to the amphoteric nature of the imidazole group, this band was mostly affected by the change in the pH of the solution. It shifts to higher energies with increasing degree of protonation of dppip-NO_2_, whereas the character of the transitions has not changed significantly. (De)protonation of the imidazole group also affects the dihedral angle between the nitrophenyl group and the imidazole–dpp ring system. However, the blue shift of the excitation energy seems to be an electrostatic effect related to the stabilization of the HOMO-3 with increasingly positive charge on the imidazole group.

In contrast to the changes in the photophysical properties, the redox potential of the Ru^II^/Ru^III^-wave of Ru(dppip-NO_2_) is comparable to Ru(dpp), differing only by 0.03 V. Likewise, both WOCs show similar catalytic performance. Interestingly, this similar performance persisted throughout the investigated pH range, despite the pH-sensitive imidazole motif of Ru(dppip-NO_2_) that possesses its p*K*_a_ value within the range of the investigated pH regime. Therefore, it can be concluded that the catalytic performance of the Ru-center was not significantly affected by the chemical modification of the dpp ligand despite the influence of the nitrophenyl group on the electronic states of the metal complex. Thus, the original catalytic properties of the parent complex were preserved. This observed independence of catalytic performance from the degree of protonation of the ligand is highly gratifying albeit not expected. Imidazole-based ligand structures in related complexes show a strong impact of the protonation state on the electronic states of the coordinated ruthenium centers.^32^ Moreover, water oxidation catalysis with molecular catalysts usually shows a susceptibility of performance to different proton concentrations.^33,34^ This holds especially true for the OEC in biological photosynthesis where ligand protonation states play a very important role in determining the overall catalytic activity.^35^ Thus, Ru(dppip-NO_2_) might serve as an active WOC capable of additionally reporting the local pH value in, e.g., polymeric materials.^36,37^

The present work can provide foundations for further developments in WOC systems. Ru(dppip-NO_2_) could serve as a WOC building block and a starting point for further modifications, such as substitution of the nitro group for an amide or azide one, which in turn could be used in click-chemistry or Schiff’s base reactions. At the same time, the presented modification strategy preserves the original catalytic properties, thus providing a WOC building block that offers a predictable catalytic behavior.

## Experimental Section

### 2-(4-Nitrophenyl)-6,9-di(pyridin-2-yl)-1*H*-imidazo[4,5-*f*][1,10]phenanthroline (dppip-NO_2_)

The free ligand was synthesized according to a modified literature procedure.^15^ A mixture of 154.5 mg of dppO_2_ (0.42 mmol, 1 equiv), 80.9 mg of 4-nitrobenzaldehyde (0.54 mmol, 1.3 equiv), and 1.45 g of ammonium acetate (18.0 mmol, 44 equiv) was suspended in 15 mL of acetic acid. The suspension was refluxed in a microwave-assisted reaction (1.5 min 600 W, 35 min 300 W). Upon cooling to room temperature, a brown precipitate formed. After diluting the solution with 45 mL of water, the pH was adjusted to pH 8 using conc. ammonia solution. Filtering off the solid and washing it with water and diethyl ether and subsequently drying it under high vacuum conditions yielded 179 mg (0.36 mmol, 85%) of dppip-NO_2_ as a brown-orange solid.

^1^H NMR (DMSO, 400 MHz,): δ 9.03 (d, *J* = 8.4 Hz, 2H), 8.98 (d, *J* = 7.9 Hz, 2H), 8.88 (d, *J* = 8.5 Hz, 2H), 8.79 (d, *J* = 3.9 Hz, 2H), 8.52 (d, *J* = 8.9 Hz, 2H), 8.46 (d, *J* = 9.1 Hz, 2H), 8.14 (td, *J* = 7.7, 1.7 Hz, 2H), 7.60–7.53 (m, 2H). HRMS/ESI (+): calcd. for C_29_H_18_N_7_O_2_ 496.15165, found 496.15122 [M + H]^+^.

### [Ru(dppip-NO_2_)(pic)_2_](PF_6_)_2_ (Ru(dppip-NO_2_)

The synthesis of Ru(dppip-NO_2_) followed a modified literature synthesis.^12,13^ In a 250 mL Schlenk flask, 100.6 mg of dppip-NO_2_ (0.22 mmol, 1 equiv) and 138.2 mg of [Ru(dmso)_4_Cl_2_] (0.29 mmol, 1.3 equiv) were suspended in 75 mL of EtOH and de-aerated for 2 h using argon. The degassed suspension was refluxed under inert conditions for 2 h before a degassed aqueous (20 mL of H_2_O) solution of 547.7 mg of LiCl (12.9 mmol, 59.8 equiv), 1.3 mL of TEA (9.3 mmol, 43.2 equiv), and 1.26 mL of 4-picoline (12.9 mmol, 43.2 equiv) was added. The resulting mixture was further refluxed under an argon atmosphere for 23 h. The obtained dark red solution was concentrated via a rotary evaporator, and the crude product was precipitated by adding an excess of NH_4_PF_6_ (∼20-fold) solved in water. The red solid was filtered off and washed with water and diethyl ether. Further purification could be obtained via diffusion crystallization from DMF and Et_2_O, yielding 66.4 mg (0.06 mmol, 29%) as a red solid.

^1^H NMR (500 MHz, DMSO) δ 10.18 (d, *J* = 5.3 Hz, 2H), 8.95 (d, *J* = 8.7 Hz, 2H), 8.82 (d, *J* = 8.8 Hz, 2H), 8.70 (d, *J* = 8.9 Hz, 2H), 8.57 (d, *J* = 7.7 Hz, 2H), 8.48 (d, *J* = 8.9 Hz, 2H), 8.27 (td, *J* = 7.8, 1.4 Hz, 2H), 8.04 (ddd, *J* = 7.3, 5.3, 1.1 Hz, 2H), 7.78 (d, *J* = 6.6 Hz, 4H), 6.88 (d, *J* = 6.6 Hz, 4H), 2.03 (s, 6H). HRMS/ESI (+): calcd. for C_41_H_31_N_9_O_2_Ru 391.58166, found 391.58189 [M-2PF_6_]^2+^, calcd. for C_41_H_30_N_9_O_2_Ru 782.15604, found 782.15666 [M-H-2PF_6_]^+^.

## Supporting Information Available

Additional details regarding the experimental and theoretical methodology and NMR, HRMS, and UV–vis spectra as well as additional details regarding the p*K*_a_ determination and the electrochemical and photocatalytic experiments (PDF)
