# Supplementary material for: A Ruthenium(II) Water Oxidation Catalyst Containing a pH-Responsive Ligand Framework
Source: Inorg Chem. 2021 Aug 10;60(17):13299–308. doi: 10.1021/acs.inorgchem.1c01646 (PMC8424647; doi:10.1021/acs.inorgchem.1c01646)
Supplement: Supplementary file 1 — ic1c01646_si_001.pdf [file ic1c01646_si_001.pdf]

Supplementary Information

Title: A Ruthenium(II) Water Oxidation Catalyst Containing a pH Responsive Ligand Framework

Authors: Fabian L. Huber,[a] Anna M. Wernbacher,[b] Daniel Perleth,[a] Djawed Nauroozi,[a] Leticia González,*[b] and Sven Rau*[a]

[a]*Institute of Inorganic Chemistry I, Ulm University, 89081 Ulm, Albert-Einstein-Allee 11, Germany*

[b]*Institute of Theoretical Chemistry, Faculty of Chemistry, University of Vienna, 1090 Vienna, Währinger Strasse 17, Austria*

Table of Contents

1. General Information S2
2. NMR Spectra S6
3. Mass Spectrometry S8
4. Theoretical evaluation of the Ru(dppip-NO_2) structure S11
5. Electronic structure S13
6. Photophysics S14
7. pK_a Determination S17
8. Electrochemistry S18
9. Catalysis S18
10. Mass spectrometry of possible catalytically active intermediates S19

References S21

1. General Information

UV-vis absorption spectroscopy: Measurements were performed at a JASCO V-670 UV-Vis Spectrophotometer using HELMA OS precision cuvettes made of quartz glass with a path length of 10 mm. Measurements were performed under aerated conditions at room temperature.

pH measurement and pK_a value determination: pH values were measure using a FiveGo pH Meter from Mettler Toledo. pK_a values were determined via UV-vis titration. **Ru(dppip-NO$_2$)** (1x10^{-5} M) was solved in a mixture of 80 v-% aqueous Britton-Robinson buffer and 20 v-% MeCN. The starting pH was 1.95 and the pH was adjusted by addition of solid NaOH between measurements. Buffer solutions for catalysis were adjusted by adding solid $NaHCO_3$ to a 0.08 M H_3BO_3 solution until the desired pH value was reached.[1]

^{1}H-NMR: Spectra were recorded on Bruker Advance 400 MHz at room temperature. The chemical shifts are given in ppm and are referenced to the corresponding solvent residual signals (2.50 ppm, quintet for DMSO; 5.32 ppm, triplet for CD_2Cl_2).

High resolution mass spectrometry (HRMS): Spectra were measured on a Bruker solariX by electron spray ionization (HRMS/ESI).

Electrochemistry: Cyclic coltammograms were measured in dimethylformamide (DMF) solution with 0.1 M $(nBu)_4NPF_6$ as supporting electrolyte. Measurements were performed with an Autolab potentistat PGSTAT204 from Metrohm using a standard three-electrode configuration with a glassy carbon working electrode, a Pt wire as counter electrode and a silver electrode as reference. After the measurements ferrocene was added to the solution and the ferrocene/ferrocenium (Fc/Fc^+) couple was used as internal reference. Thus, all potentials are reported versus Fc/Fc^+.

Oxygen detection: O_2 concentrations were measured using a FireStingO2 optical oxygen meter (Pyroscience, Germany) using oxygen sensitive optical sensor spots (OXSP5, with optical isolation). The spot was glued (transparent silicone glue, SPGLUE). to the inner glass wall of a screw-capped vial. The O2 concentration was measured in µmol/L (solution) and mbar (gas-phase). Two-point calibration of the liquid phase was performed using a de-oxygenated aqueous solution (aqueous sodium dithionite solution) and air equilibrated deionized water. Two-calibration of the gas-phase was performed against Ar-atmosphere and ambient air. Solution turn over numbers (TONs) were calculated based on the detected concentration, gas-phase TONs were calculated utilizing the ideal gas equation. This method of oxygen detection has been reported previously.[2]

Irradiation setup: Irradiation of the samples was performed via one LED-stick (λ_{max} = 470 nm, 45-50 mW cm^{-2}). During irradiation reaction vessels were tempered by a custom air cooling setup (25 °C). To ensure reproducible irradiation conditions, the reaction vial and LED-stick were fixated with a 3D-printed holder (also see Figure S1).

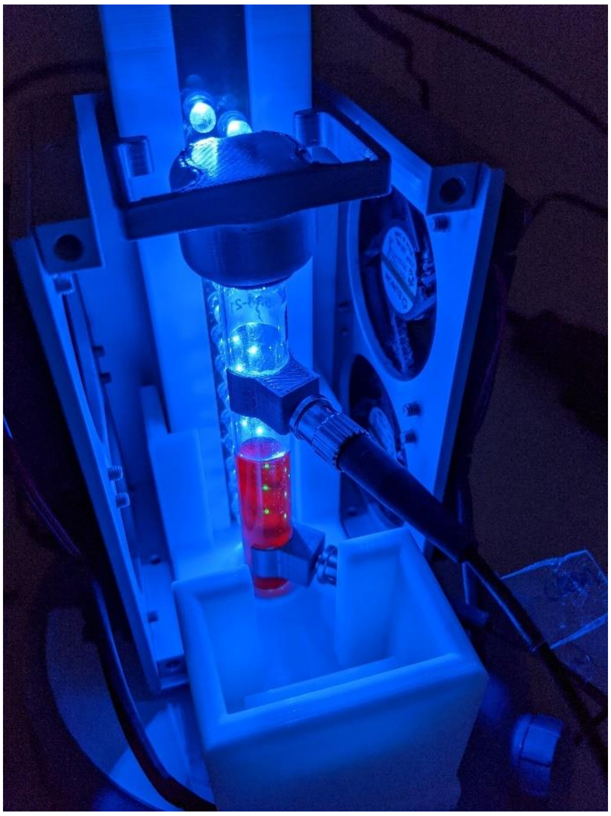

Figure S1: Depiction of the experimental setup of light driven water oxidation.

Photochemical water oxidation catalysis: For photocatalytical studies, 2.6 µM catalysts (Ru(dpp) or Ru(dppip-NO_2), 0.3 mM PS and 10 mM $Na_2S_2O_8$ were solved in a mixture consisting of 96 v-% aqueous H_3BO_3/$NaHCO_3$ buffer (0.08 M H_3BO_3) and 4 v-% MeCN. All experiments were carried out in de-aerated solvents under argon atmosphere. During the reaction the solution was stirred continuously.

Chemical oxidation of Ru(dppip-NO_2): Chemical oxidation of Ru(dppip-NO_2) was performed as previously reported.[3] To a 1.0 mM solution of Ru(dppip-NO_2) in water containing 4 vol% MeCN and 0.01 M HNO_3 different amounts of $(NH_4)_2[Ce(NO_3)_6]$ (CAN; 4 eq., 8 eq. and 12 eq.) were added. The solutions were then stirred for 20 min at room temperature before the solvent was evaporated. Afterwards the samples were analyzed by electrospray ionization mass spectrometry (ESI-MS) measurements on an Advion expression-L CMS with a single quadrupole mass analyser and an electron multiplier with a high energy conversion dynode detector. The unit mass resolution of 0.5 to 0.7 m/z (FWHM) is provided over the range of 0 – 2000 m/z units and the accuracy is 0.1 m/z.

Chemicals and synthetic procedures: Commercially available chemicals were used as received. All solvents of technical grade were redistilled prior to use. Solvents of higher quality were used without further purification. All reactions were carried out under ambient laboratory conditions, if not stated otherwise. For synthesis under inert conditions standard Schlenk techniques were applied with argon as inert gas. 2,9-di(pyridine-2-yl)-1,10-phenanthroline-5,6-dione (dppO_2),[4,5] $[Ru(dpp)(pic)_2](PF_6)_2$ (Ru(dpp))[5] and $[(dceb)_2Ru(bpy)](PF_6)_2$ (PS)[6] were synthesized according to literature known procedures.

Computational Methods: All density functional theory (DFT) and time-dependent DFT (TDDFT) calculations were performed with the ORCA 4.2[7] program package using B3LYP[8–11]-D3BJ[12,13], the C-PCM[14] continuum solvation model (acetonitrile) and using the RIJCOSX[15–17] approximation for computational efficiency[18] together with the SARC/J[19,20] auxiliary basis set. B3LYP was chosen since it is reported to yield good results for computing absorption spectra of Ru-complexes.[21–26] The geometries were optimized with DFT using the def2-TZVP[27] basis set with the corresponding effective core potential (def2-ECP)[28] on Ru, and for C-PCM the Gaussian charge scheme[29] was used as implemented in ORCA 4.2. Frequency calculations showed that the optimized structures correspond to minima (no imaginary frequency). TDDFT in the Tamm-Dancoff approximation (TDA)[30] was used to compute the spectra with the scalar relativistic ZORA[31] Hamiltonian, and the relativistically recontracted versions of the basis sets,[32] ZORA-def2-TZVP and ZORA-TZVP on Ru. In the case of Ru(dpp), 150 singlet excited states were calculated, and 200 states for Ru(dppip-NO_2).

The stick spectra of the equilibrium geometries were convoluted with Gaussian functions employing a full width at half maximum (fwhm) of 0.35 eV to obtain their absorption spectra. Furthermore, 50 geometries for each Ru-complex were sampled[33] from a temperature-dependent Wigner distribution[34,35] at 300 K excluding low-frequency vibrational modes below 40 cm^{-1} (50 cm^{-1} for 0H-Ru(dppip-NO_2)). For the corresponding spectra, Gaussian functions with a fwhm of 0.30 eV were used.

An automatized charge transfer analysis of the transition density matrix was performed with the TheoDORE[36–38] package. For this, the Ru-complexes were divided into chromophoric fragments as depicted in Figure S2 based on a hierarchical clustering ansatz[39] (Ru(dppip-NO_2): 1. Ru, 2. dppi fragment, 3. pic ligands, 4. nitrophenyl fragment; Ru(dpp): 1. Ru, 2. dpp ligand, 3. pic ligands).

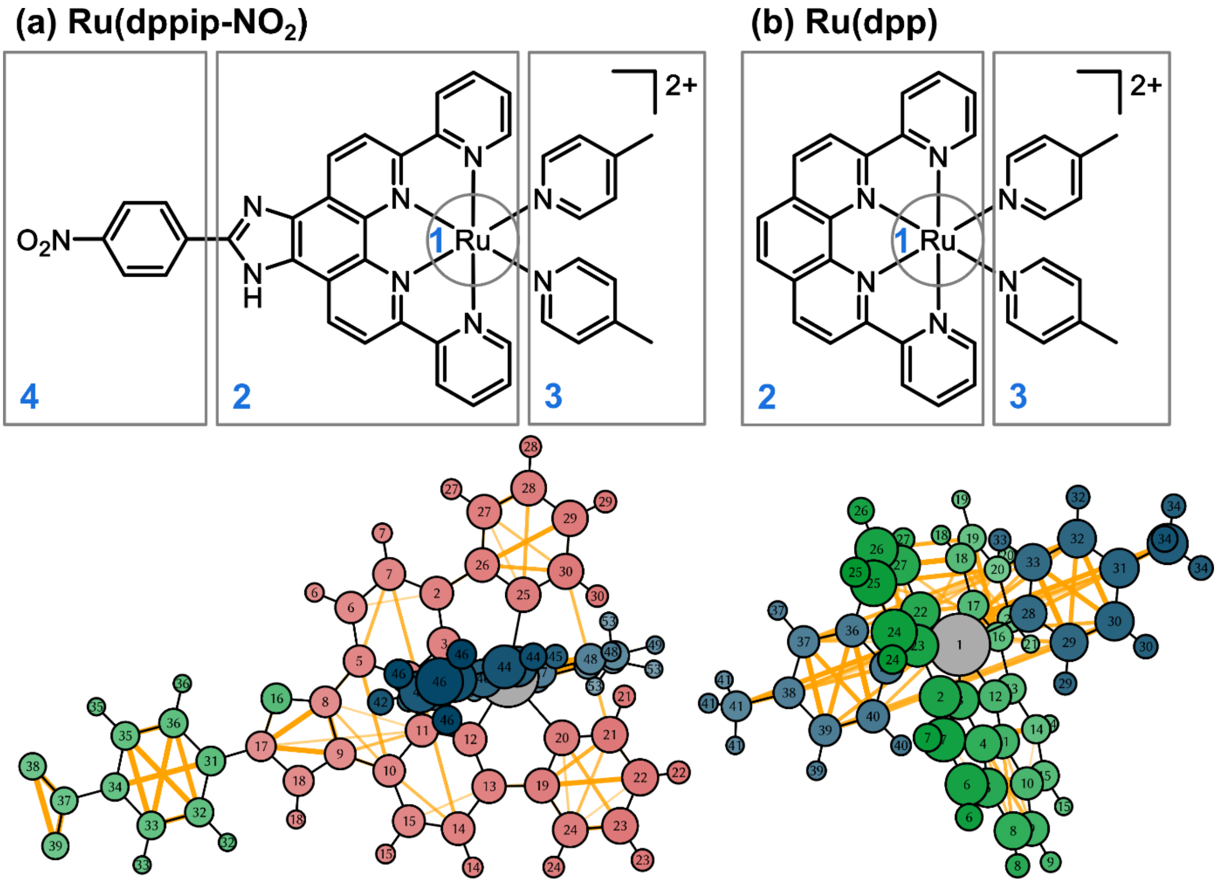

Figure S2: Fragmentation of Ru-complexes for charge transfer analysis with TheoDORE. **a** Four fragments of Ru(dppip-NO_2): 1. Ru, 2. dppi fragment, 3. pic ligands, 4. nitrophenyl fragment; **b** three fragments of Ru(dpp): 1. Ru, 2. dpp ligand, 3. pic ligands.

The pH-dependent spectra of the Ru-complex were calculated using the B3LYP spectra from the 300 K Wigner ensembles of 1H-Ru(dppip-NO_2) and 2H-Ru(dppip-NO_2). The intensities $\mathrm{Int}_{\mathrm{Ru1H,calc.}}$ and $\mathrm{Int}_{\mathrm{Ru2H,calc.}}$ were scaled based on the relative fractional concentrations α_{Ru1H} and α_{Ru2H} of the two species at different pH values using the experimental $\mathrm{pK}_{a,1}$ of 6.8.

$$\mathrm{Ru2H} \leftrightharpoons \mathrm{Ru1H} + \mathrm{H}^{\oplus} \qquad K_{a,1} = \frac{[\mathrm{H}^{\oplus}][\mathrm{Ru1H}]}{[\mathrm{Ru2H}]} = 10^{-\mathrm{pK}_{a,1}}$$

$$\mathrm{Int}_{\mathrm{Ru2H}}(\mathrm{pH}) = \alpha_{\mathrm{Ru2H}} \cdot \mathrm{Int}_{\mathrm{Ru2H,calc.}} = \left(\frac{10^{-\mathrm{pH}}}{10^{-\mathrm{pH}} + 10^{-\mathrm{pK}_{a,1}}}\right) \cdot \mathrm{Int}_{\mathrm{Ru2H,calc.}}$$

$$\mathrm{Int}_{\mathrm{Ru1H}}(\mathrm{pH}) = \alpha_{\mathrm{Ru1H}} \cdot \mathrm{Int}_{\mathrm{Ru1H,calc.}} = \left(\frac{10^{-\mathrm{pK}_{a,1}}}{10^{-\mathrm{pH}} + 10^{-\mathrm{pK}_{a,1}}}\right) \cdot \mathrm{Int}_{\mathrm{Ru1H,calc.}}$$

The (relative) total intensity as a function of the pH is given by the sum of $\mathrm{Int}_{\mathrm{Ru1H}}(\mathrm{pH})$ and $\mathrm{Int}_{\mathrm{Ru2H}}(\mathrm{pH})$. This yields the spectrum of 1H-Ru(dppip-NO_2) at pH >> $\mathrm{pK}_{a,1}$ ($\alpha_{\mathrm{Ru1H}} \approx 1$) and the spectrum of 2H-Ru(dppip-NO_2) at pH << $\mathrm{pK}_{a,1}$ ($\alpha_{\mathrm{Ru2H}} \approx 1$), while the spectrum at pH = $\mathrm{pK}_{a,1}$ corresponds to a 50-50 mixture of the two species ($\alpha_{\mathrm{Ru1H}} = \alpha_{\mathrm{Ru2H}} = 0.5$).

2. NMR Spectra

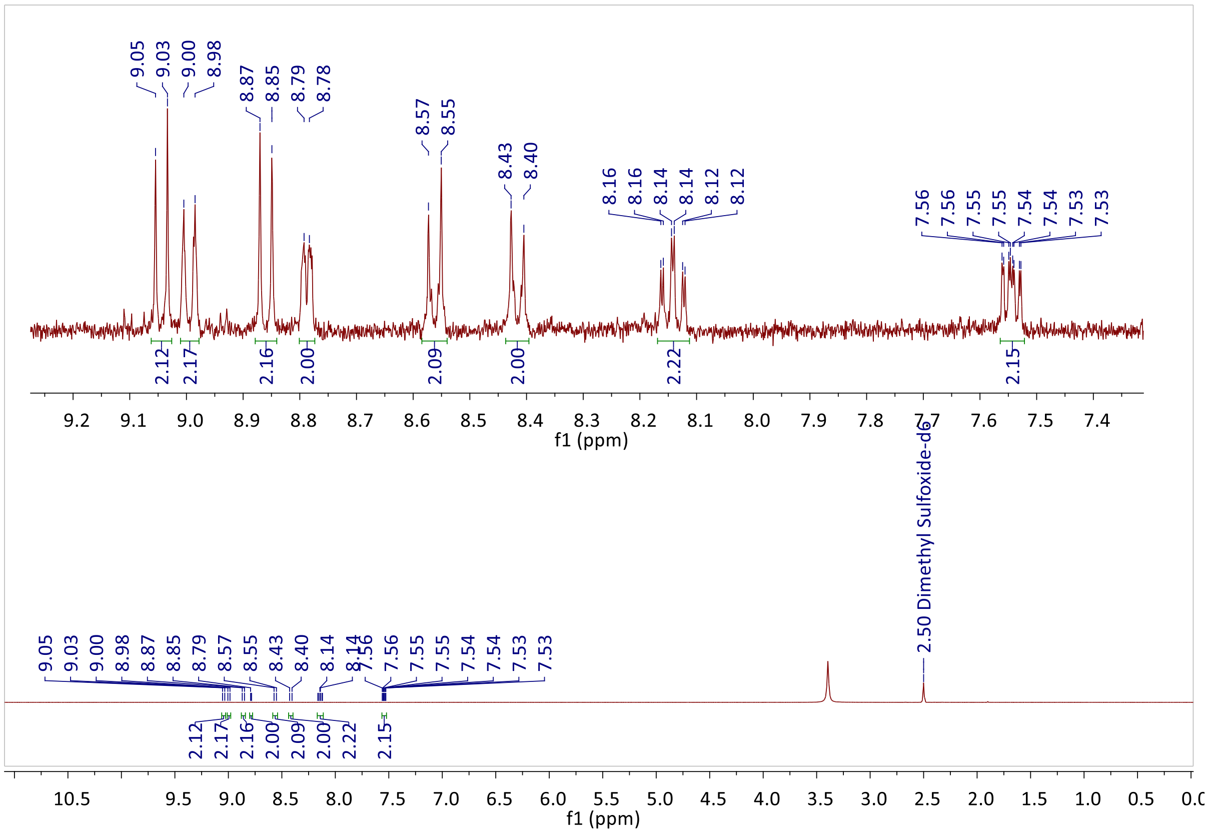

Figure S3: ^{1}H-NMR spectrum of dppip-NO_2 measured in deuterated DMSO. The aromatic section of the spectrum is enhanced.

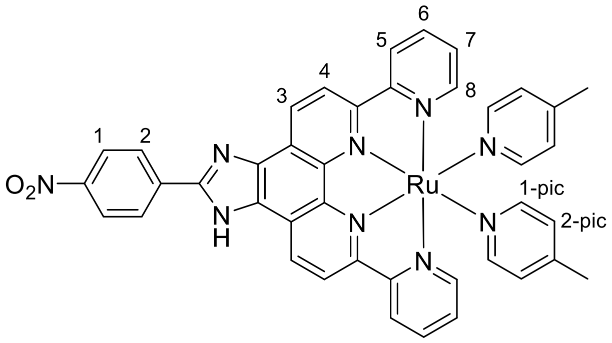

Figure S4: Schematic structure of Ru(dppip-NO_2). Signal notation used in ^{1}H-NMR spectra is given.

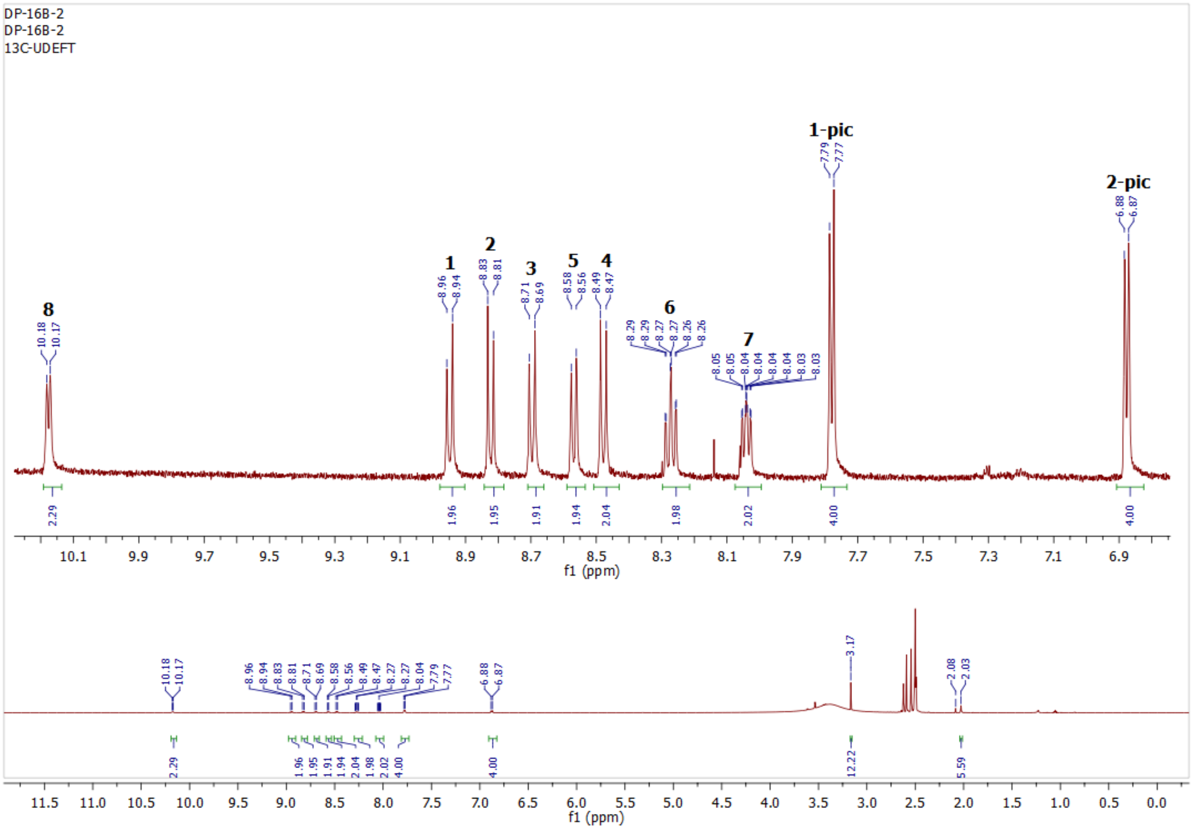

Figure S5: ^{1}H-NMR spectrum of Ru(dppip-NO_2) measured in deuterated DMSO. The aromatic section of the spectrum is enhanced.

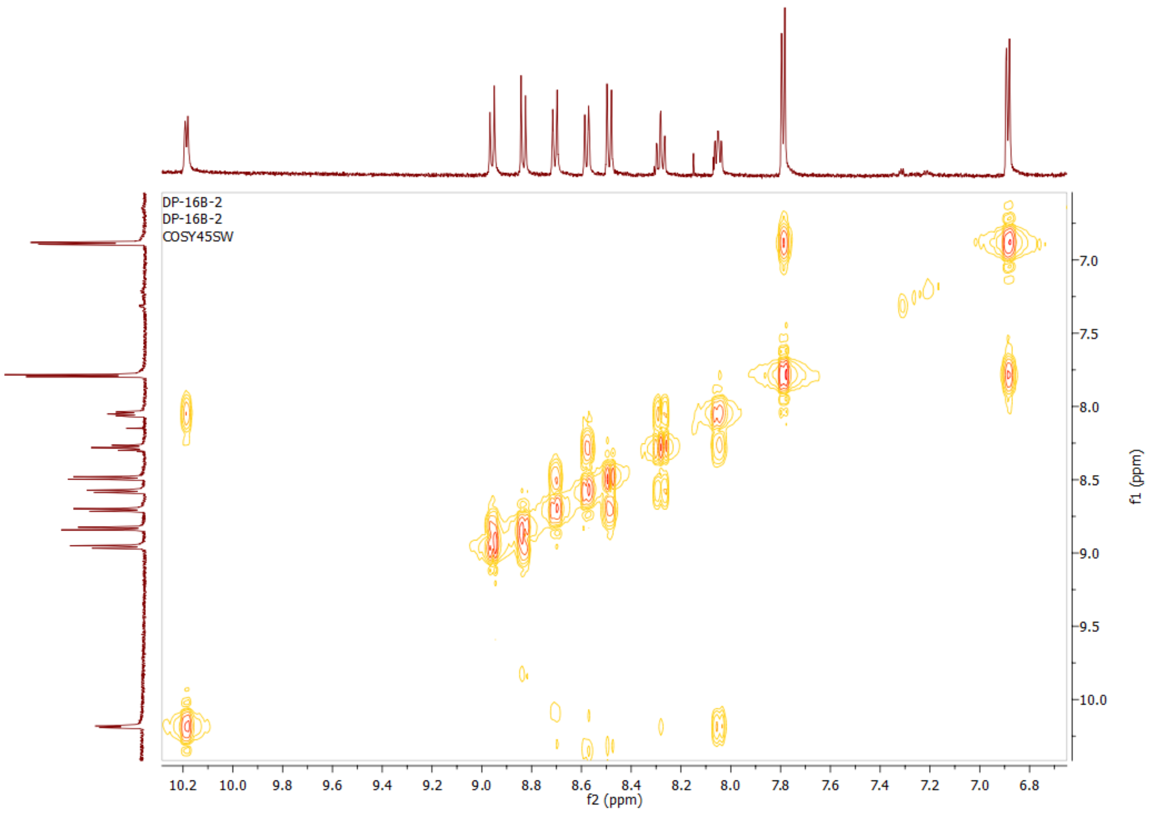

Figure S6: Aromatic region of the H,H-COSY spectrum of Ru(dppip-NO_2) measured in deuterated DMSO.

3. Mass Spectrometry

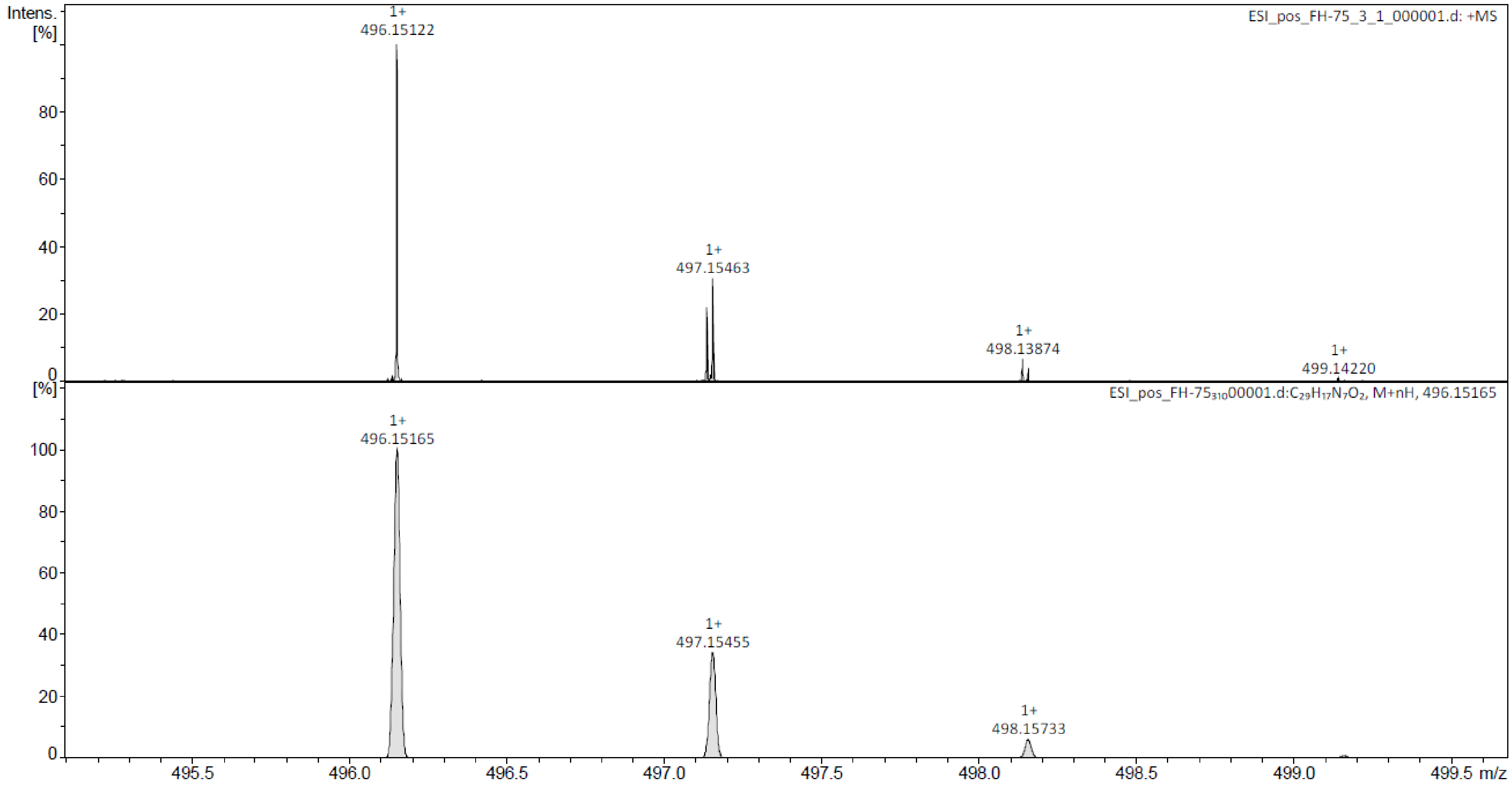

Figure S7: measured HRMS/ESI spectrum (top) and simulated mass spectrum of dppip-NO_2 (bottom).

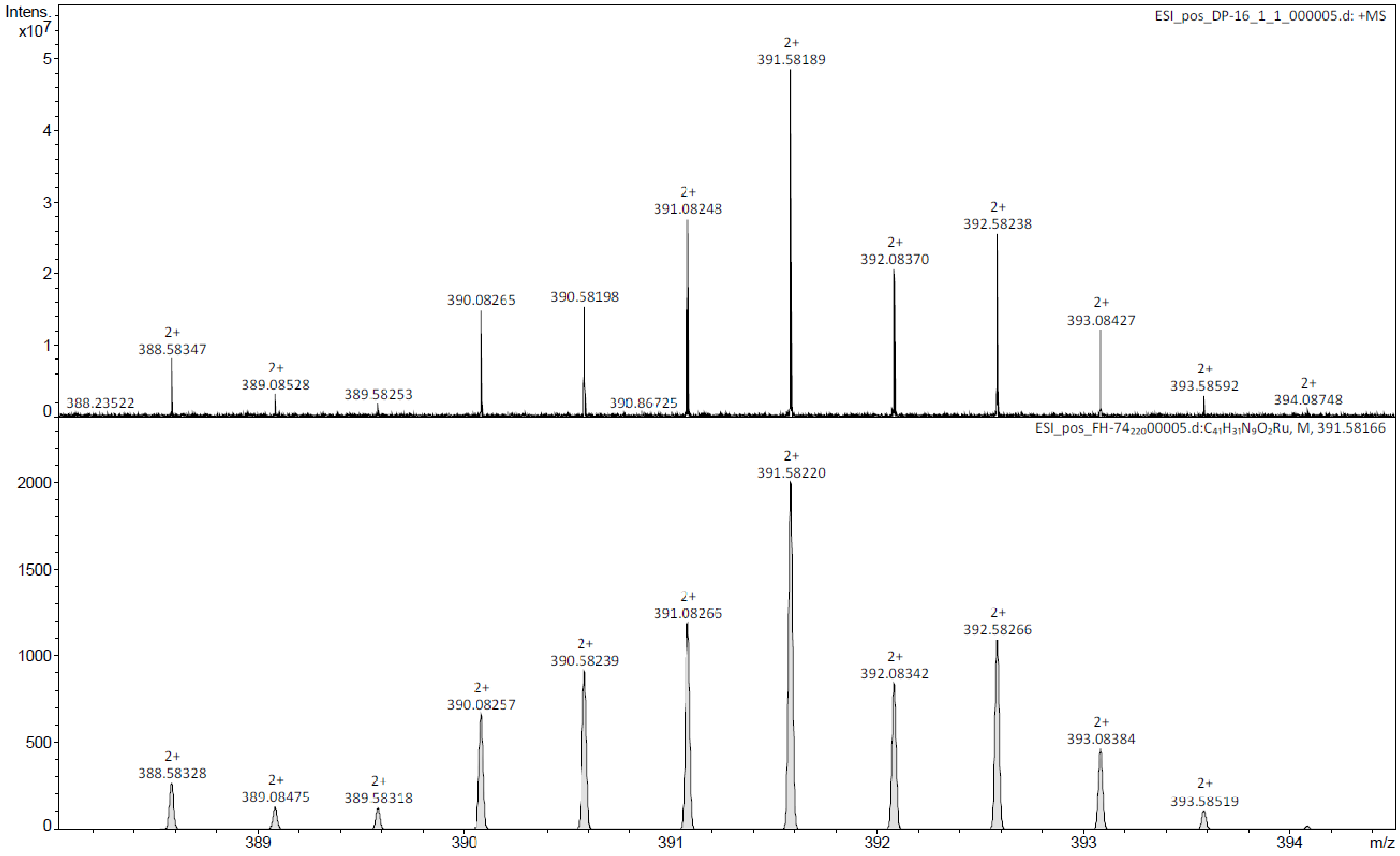

Figure S8: measured HRMS/ESI spectrum (top) and simulated mass spectrum of Ru(dppip-NO_2) (bottom) around 391 m/z.

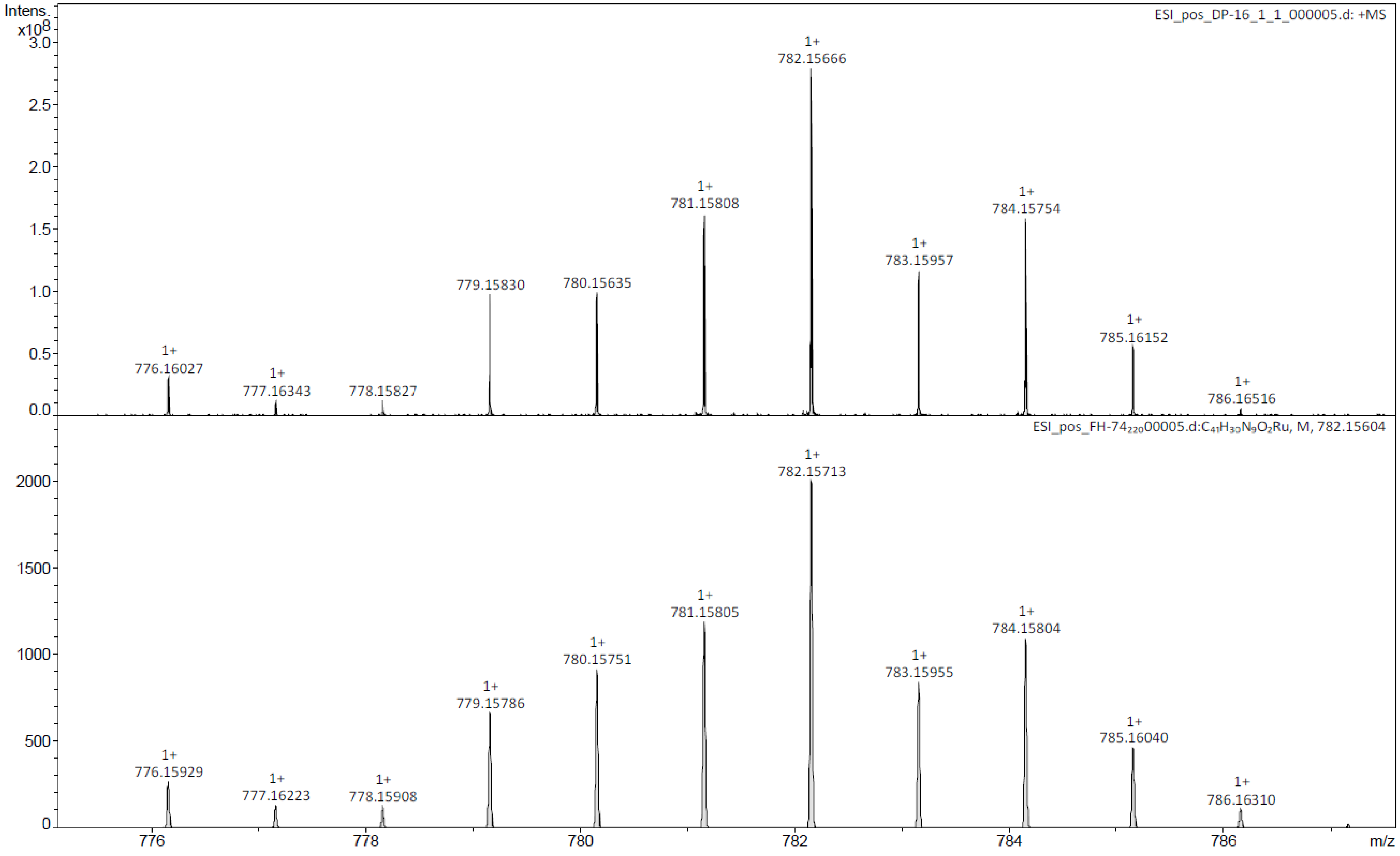

Figure S9: measured HRMS/ESI spectrum (top) and simulated mass spectrum of Ru(dppip-NO_2) (bottom) around 782 m/z

4. Theoretical evaluation of the Ru(dppip-NO_2) structure

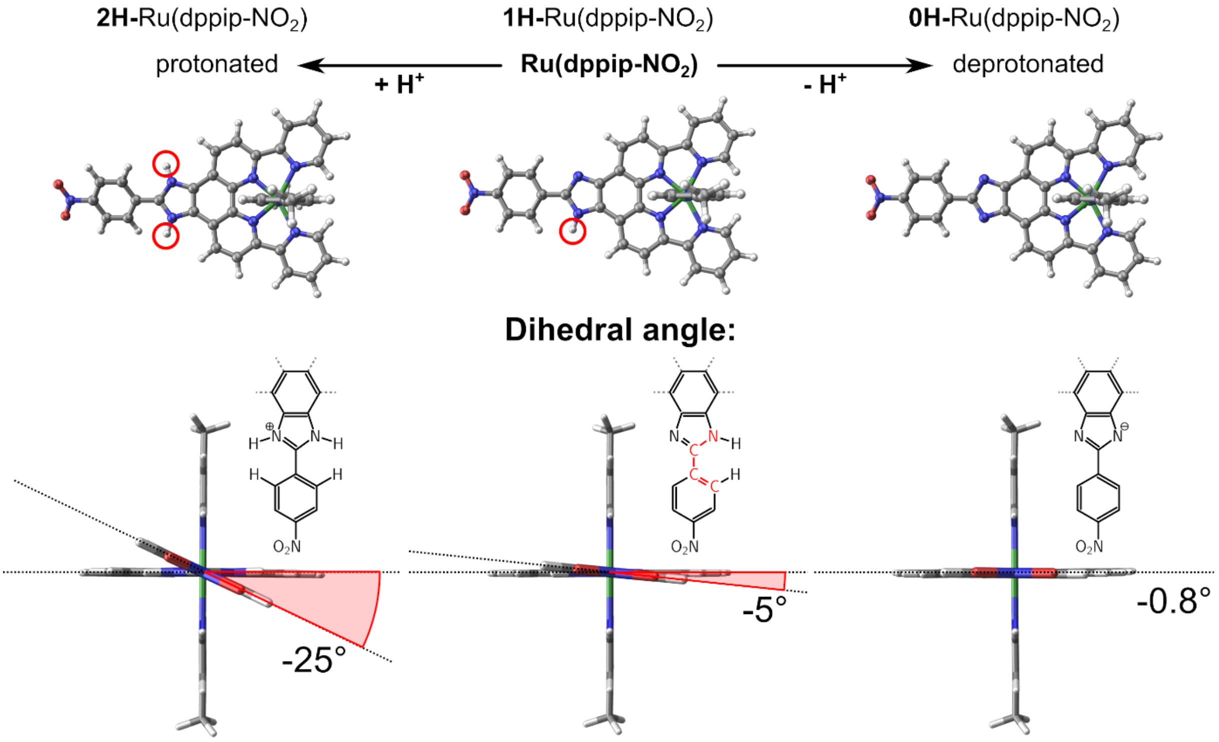

Figure S10: Calculated optimized geometries of protonated 2H-Ru(dppip-NO_2), 1H-Ru(dppip-NO_2), and deprotonated 0H-Ru(dppip-NO_2) complexes showing changes in the dihedral angle between nitrophenyl group and imidazole-dpp ring.

Table S1: Selected calculated structural parameters of 1H-Ru(dppip-NO_2) ([Ru(dppip-NO_2)(pic)$_2$]$^{2+}$) and its protonated (2H) and deprotonated (0H) forms and comparison to Ru(dpp) ([Ru(dpp)(pic)$_2$]$^{2+}$) including the experimental X-ray crystal structure data of Ru(dpp). (B3LYP-D3BJ, def2-TZVP and def2-ECP on Ru, C-PCM (MeCN)).

	**Computed**				**Exp. X-ray[a]**
	**Ru(dppip-NO$_2$)**			**Ru(dpp)**	**Ru(dpp)**
	**0H**	**1H**	**2H**		
	**Bond lengths / Å**				
**Ru-N(pic)**	2.10	2.10	2.10	2.10	2.10
**Ru-N(dpp)** (central phen)	1.96	1.96	1.96	1.96	1.94
**Ru-N(dpp)** (peripheral py)	2.18	2.18	2.17	2.19	2.17
**N(dpp)-N(dpp)** (py)	3.86	3.86	3.86	3.90	3.86
	**Bond angles / °**				
**N(pic)-Ru-N(pic)**	174	173	173	173	170
**N(dpp)-Ru-N(dpp)** (phen)	82	82	82	82	82.3
**N(dpp)-Ru-N(dpp)** (py)	125	125	125	126	125.6

**N(dpp)-Ru-N(dpp)** (phen/py)	77	76	76	76	76.1
	**Dihedral angles / °**				
**N-N-N-N (dpp)**	0.0	0.1	0.1	0.0	-0.2
**C-C-C-N** (nitrophen-im(dpp))	-1	-5	-25	/	/

[a] from refs.[5,40]

5. Electronic structure

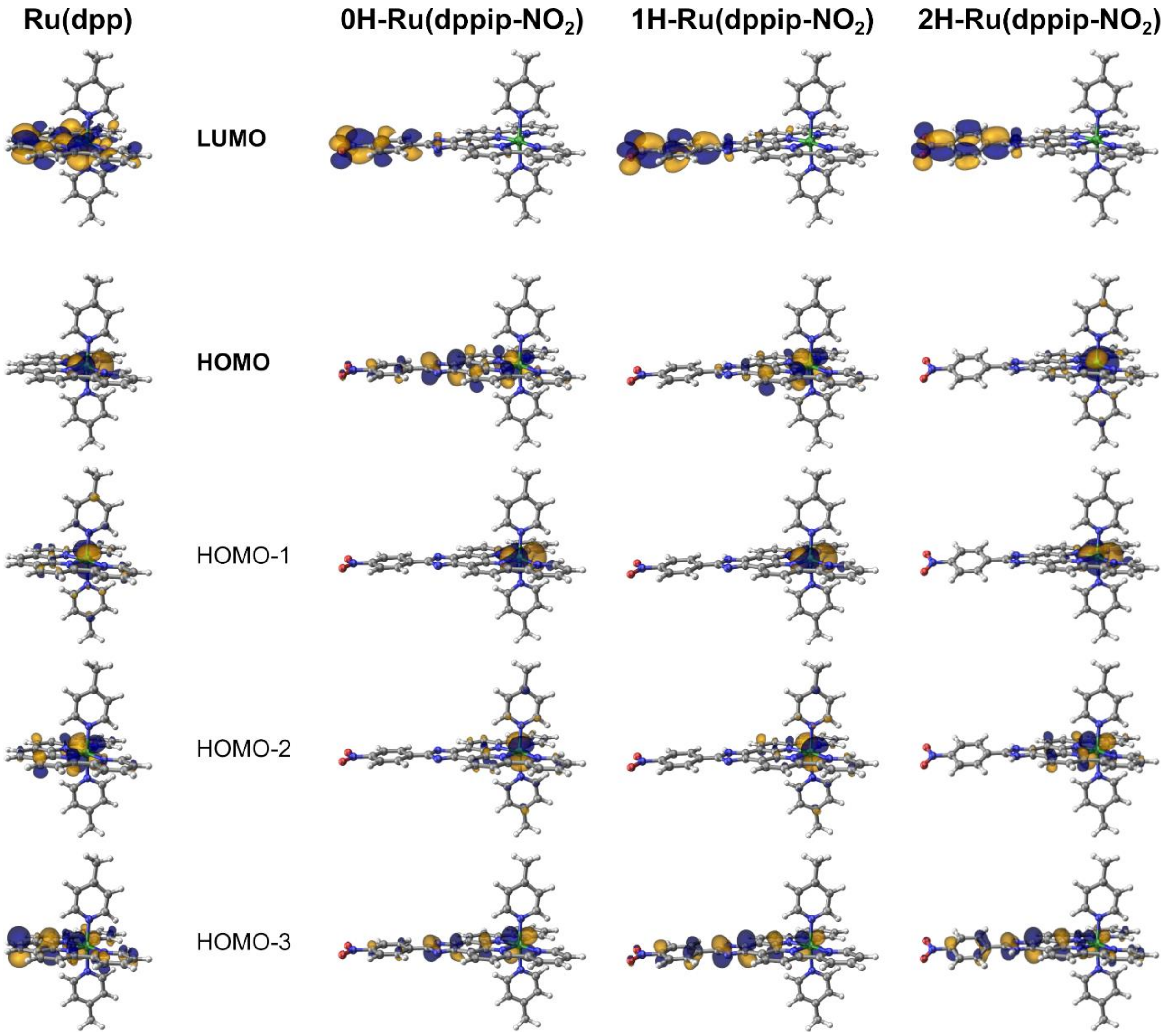

Figure S11: Highest occupied (HOMOs) and lowest unoccupied molecular orbitals (LUMO) of Ru(dpp) and of 1H-Ru(dppip-NO_2) and its deprotonated (0H) and protonated (2H) forms.

6. Photophysics

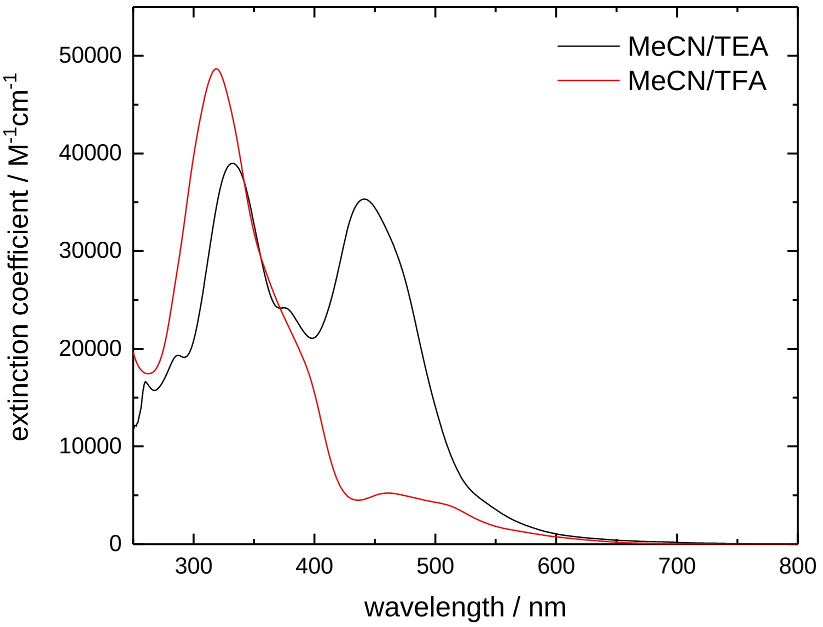

Figure S12: Extinction coefficients of Ru(dppip-NO_2) in MeCN with 0.25 v-% trimethylamine (TEA) or trifluoroacetic acid (TFA) respectively.

Extinction coefficients have been determined at $0.5 \cdot 10^{-5}$ M, $1 \cdot 10^{-5}$ M and $2 \cdot 10^{-5}$ M, which have then been averaged. All measurements were conducted three times.

Figure S13 compares the computed equilibrium spectra of Ru(dpp) and 1H-Ru(dppip-NO_2). The contribution of a specific fragment to the total spectrum was analyzed with TheoDORE. States with a significant contribution of the nitrophenyl group in 1H-Ru(dppip-NO_2), as specified by a sum of the charge transfer (CT) numbers on the nitrophenyl fragment equal to or greater than 0.33, were convoluted with Gaussians to the red sub-spectrum in Figure S13. The residual states, which only have a minor or no contribution from the nitrophenyl group, were combined to the "rest" sub-spectrum shown in blue. Interestingly, this residual spectrum of 1H-Ru(dppip-NO_2) in blue between ca. 275 and 700 nm closely resembles the spectrum of Ru(dpp) showing an intense band at 298 nm with a shoulder at ca. 330 nm (compared to 306 and 334 nm in Ru(dpp), cf. Table S3), followed by two smaller bands in the visible region at 398 nm and 513 nm (412 and 517 nm in Ru(dpp)).

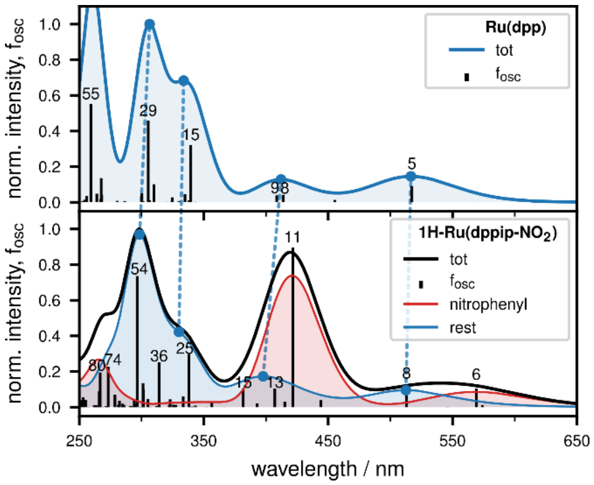

Figure S13: Calculated absorption spectrum and oscillator strengths f_{osc} of Ru(dpp) compared to 1H-Ru(dppip-NO$_2$). States with significant contribution of nitrophenyl group in Ru(dppip-NO$_2$) (ΣCT numbers $\geq$ 0.33) are convoluted to the red sub-spectra, the other states to the blue sub-spectra (Gaussian line shape, fwhm of 0.35 eV). (B3LYP-D3BJ, ZORA, ZORA-def2-TZVP and ZORA-TZVP on Ru, C-PCM (MeCN)).

The excitation energies, oscillator strengths (f_{osc}) and main excited-state characters of intense transitions computed for the equilibrium structures of the Ru-catalysts are summarized in Table S2 and Table S3 below and compared to experimental values.

Table S2: Selected intense states of 1H-Ru(dppip-NO$_2$) and its protonated ("2H") and deprotonated forms ("0H"): vertical excitation energies E, wavelengths λ, oscillator strengths f_{osc}, and main state characters; wavelengths λ_{max} of peak maxima or shoulders "sh" in computed convoluted spectra (Gaussian line shape, fwhm of 0.35 eV) and comparison to experimental absorption peak maxima of Ru(dppip-NO$_2$) in MeCN/TFA and MeCN/TEA. (B3LYP-D3BJ, ZORA, ZORA-def2-TZVP and ZORA-TZVP on Ru, C-PCM (MeCN)).

**2H-Ru(dppip-NO$_2$)**						**Exp. in MeCN/TFA**
**State**	**E / eV**	**λ / nm**	**f_{osc}**	**Main character**	**λ_{max} / nm**	**Exp. λ_{max} / nm**
3	1.74	713	0.05	Ru → im-nitrophenyl MLCT	712	510 sh
8	2.39	519	0.08	Ru → dpp MLCT	517	462
**13**	**3.15**	**394**	**0.94**	**(Ru)-im-dpp-phenyl → nitrophenyl LLCT**	**394**	**383 sh**
31	3.76	330	0.32	Ru → pic MLCT		
**53**	**4.05**	**306**	**0.46**	**dpp → nitrophenyl LLCT (+ LC)**	**311**	**319**
**54**	**4.07**	**305**	**0.22**	**dpp → dpp, pic, nitrophenyl LLCT + LC (+ MLCT)**		
76	4.55	272	0.68	dpp → dpp LC (+ LLCT, MLCT)	274 (sh)	
**1H-Ru(dppip-NO$_2$)**						**Exp. in MeCN/TEA**
**State**	**E / eV**	**λ / nm**	**f_{osc}**	**Main character**	**λ_{max} / nm**	**Exp. λ_{max} / nm**
6	2.18	569	0.10	Ru → dppip-NO$_2$ MLCT	540	/
8	2.42	513	0.11	Ru → dppip-NO$_2$ MLCT		
**11**	**2.94**	**421**	**0.90**	**Ru-im-dpp-(phenyl) → nitrophenyl**	**419**	**441**

				**LLCT + MLCT**		
25	3.67	338	0.29	Ru → pic<br>MLCT	330 sh	377 sh
**54**	**4.19**	**296**	**0.74**	**(Ru)-dpp → dpp<br>LC (+ LLCT, MLCT)**	**298**	**332**
74	4.54	273	0.23	dpp → dpp<br>LC + MLCT + LLCT	270 sh	287
80	4.65	267	0.20	Ru-im-dpp-(phenyl) → dpp<br>LLCT + LC + MLCT		
**0H-Ru(dppip-NO_2)**						
**State**	**E / eV**	**λ / nm**	**f_{osc}**	**Main character**	**$λ_{max}$ / nm**	
2	1.95	637	0.45	Ru-im-dpp → nitrophenyl<br>LLCT + MLCT	635	
**10**	**2.66**	**465**	**0.34**	**Ru-im-dpp → nitrophenyl<br>MLCT + LLCT**	**458**	
**12**	**2.77**	**448**	**0.39**	**Ru → dpp<br>MLCT**		
28	3.55	349	0.31	Ru → pic<br>MLCT	**325**	
**42**	**3.86**	**321**	**0.64**	**Ru-dpp → dpp<br>LC + MLCT (+ LLCT)**		
71	4.37	284	0.31	dpp → dpp, nitrophenyl<br>LLCT + LC (+ MLCT)	285 (sh)	

Table S3: Selected intense states of Ru(dpp): vertical excitation energies E, wavelengths λ, oscillator strengths f_{osc}, and main state characters; wavelengths $λ_{max}$ of peak maxima or shoulders "sh" in computed convoluted spectra (Gaussian line shape, fwhm of 0.35 eV). (B3LYP-D3BJ, ZORA, ZORA-def2-TZVP and ZORA-TZVP on Ru, C-PCM (MeCN)).

**Ru(dpp)**					
**State**	**E / eV**	**λ / nm**	**f_{osc}**	**Main character**	**$λ_{max}$ / nm**
5	2.40	517	0.09	Ru → dpp MLCT	517
8	3.00	414	0.04	Ru → dpp MLCT	412
9	3.04	408	0.04	Ru → dpp MLCT	
15	3.65	339	0.32	Ru → pic MLCT	334 sh
**29**	**4.06**	**305**	**0.46**	**dpp → dpp LC**	**306**

7. pK_a Determination

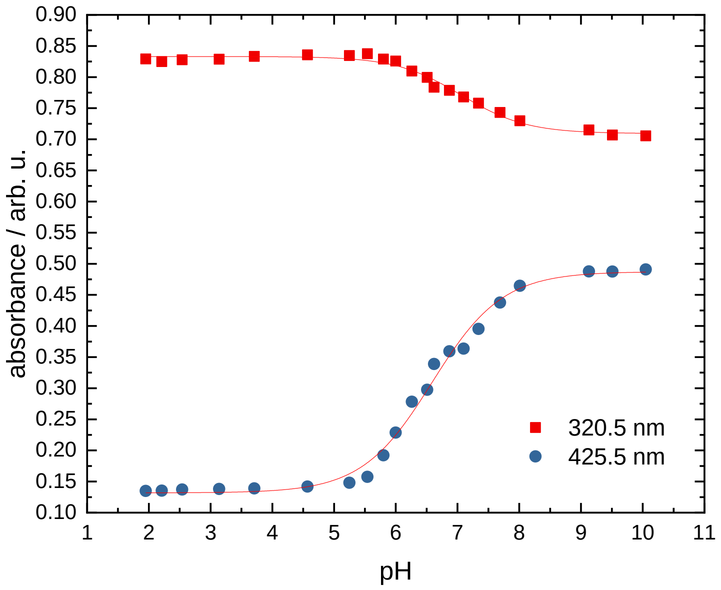

Figure S14: Absorbance of Ru(dppip-NO_2) at 320.5 nm (red squares) and 425.5 nm (blue dots) as a function of pH. The red curves are a sigmoidal fit.

8. Electrochemistry

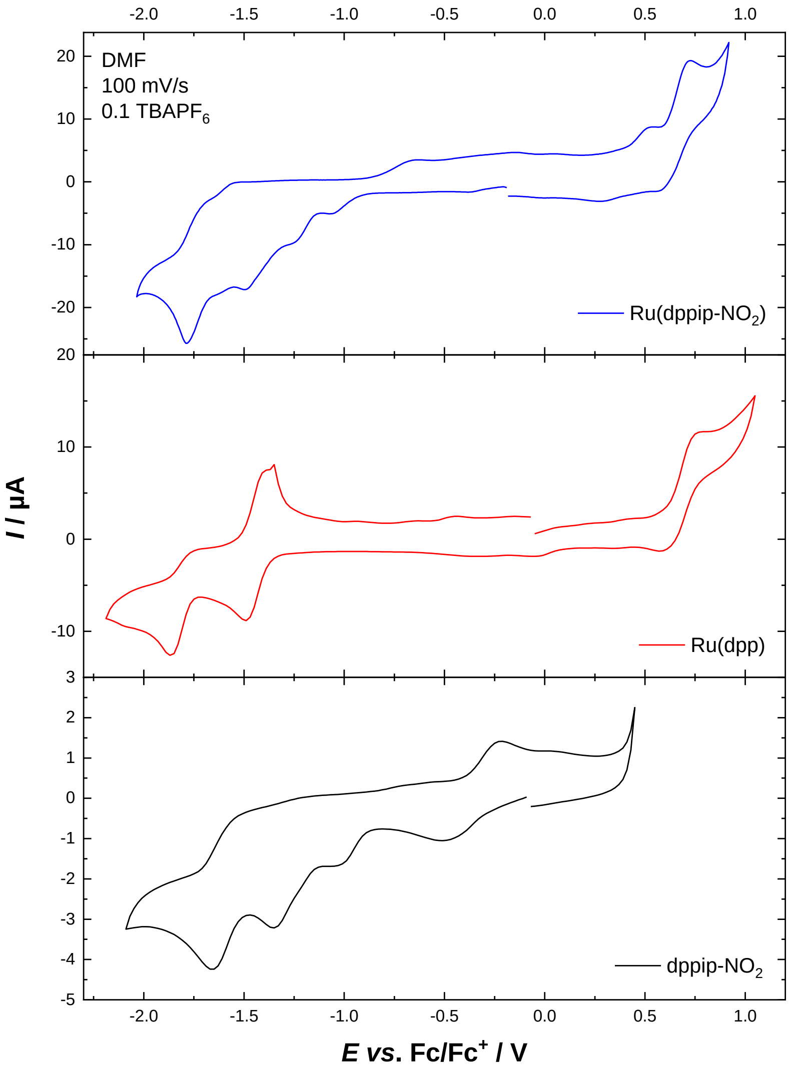

Figure S15 Cyclic voltammograms of Ru(dppip-NO_2), Ru(dpp) and dppip-NO_2 in DMF containing 0.1 M $(nBu)_4NPF_6$ as supporting electrolyte. The measurements were performed at 100 mV s^{-1} and referenced versus Fc/Fc^+.

9. Catalysis

Turnover number:

$$TON = \frac{n(O_2)}{n(catalyst)}$$

Turnover frequency:

$$TOF = \frac{TON}{t}$$

10. Mass spectrometry of possible catalytically active intermediates

1.) No addition of $(NH_4)_2[Ce(NO_3)_6]$:

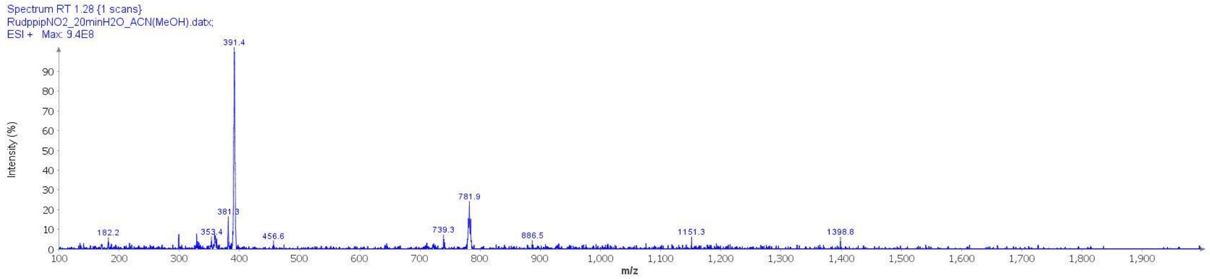

Figure S16: ESI(+) mass spectrum of Ru(dppi-NO_2) with previous treatment with $(NH_4)_2[Ce(NO_3)_6]$.

Table S4: Peak assignment of the mass spectrum depicted in Figure S16.

Peak	Assignment
781.9	$[M\text{-}2PF_6^{-}\text{-}H]^{+}$
391.4	$[M\text{-}2PF_6^{-}]^{2+}$

2.) Addition of 4 eq. $(NH_4)_2[Ce(NO_3)_6]$:

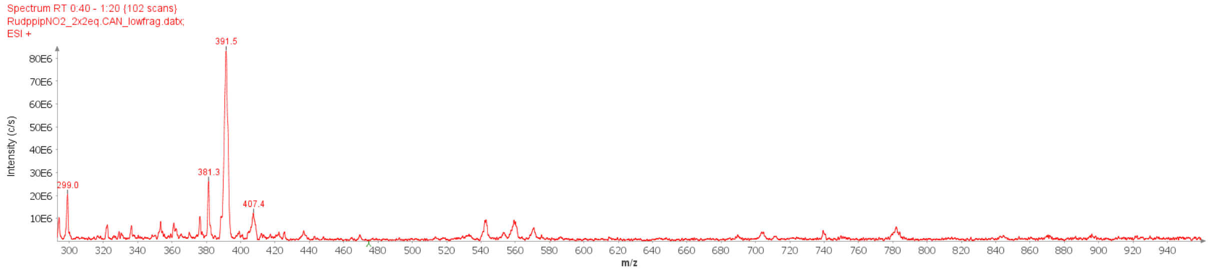

Figure S17: ESI(+) mass spectrum of Ru(dppi-NO_2) after treatment with 4 eq. $(NH_4)_2[Ce(NO_3)_6]$.

Table S5: Peak assignment of the mass spectrum depicted in Figure S17.

Peak	Assignment
407.4	$[M\text{-}2PF_6^{-}+2O]^{2+}$
391.5	$[M\text{-}2PF_6^{-}]^{2+}$

3.) Addition of 8 eq. $(NH_4)_2[Ce(NO_3)_6]$:

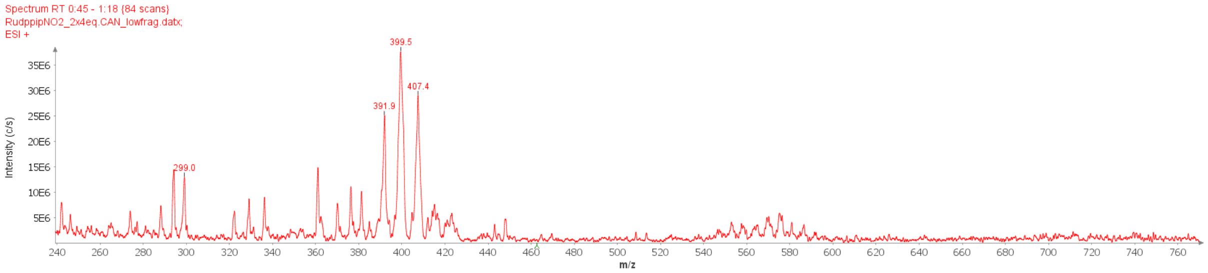

Figure S18: ESI(+) mass spectrum of Ru(dppi-NO_2) after treatment with 8 eq. $(NH_4)_2[Ce(NO_3)_6]$.

Table S6: Peak assignment of the mass spectrum depicted in Figure S18.

Peak	Assignment
423.0	$[M-2PF_6^-+2O+MeO]^{2+}$
415.1	$[M-2PF_6^-+O+MeO]^{2+}$
407.4	$[M-2PF_6^-+2O]^{2+}$
399.5	$[M-2PF_6^-+O]^{2+}$
391.9	$[M-2PF_6^-+H]^{2+}$
376.3	$[M-2PF_6^--Pic+2O+MeOH]^{2+}$

4.) Addition of 12 eq. $(NH_4)_2[Ce(NO_3)_6]$:

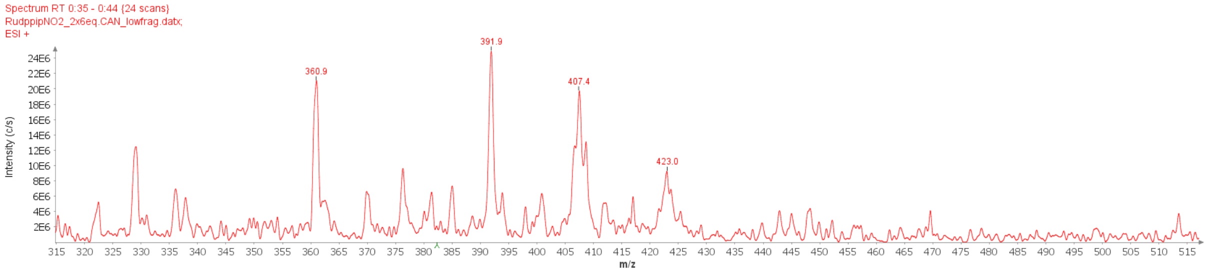

Figure S19: ESI(+) mass spectrum of Ru(dppi-NO_2) after treatment with 12 eq. $(NH_4)_2[Ce(NO_3)_6]$.

Table S7: Peak assignment of the mass spectrum depicted in Figure S19.

Peak	Assignment
423.0	$[M-2PF_6^-+2O+MeO]^{2+}$
407.4	$[M-2PF_6^-+2O]^{2+}$
391.9	$[M-2PF_6^-+H]^{2+}$
376.3	$[M-2PF_6^--Pic+2O+MeOH]^{2+}$

References

(1) Okamura, M.; Yoshida, M.; Kuga, R.; Sakai, K.; Kondo, M.; Masaoka, S. A Mononuclear Ruthenium Complex Showing Multiple Proton-Coupled Electron Transfer toward Multi-Electron Transfer Reactions. *Dalt. Trans.* **2012**, *41* (42), 13081–13089. https://doi.org/10.1039/c2dt30773a.

(2) Huber, F. L.; Amthor, S.; Schwarz, B.; Mizaikoff, B.; Streb, C.; Rau, S. Multi-Phase Real-Time Monitoring of Oxygen Evolution Enables *in Operando* Water Oxidation Catalysis Studies. *Sustain. Energy Fuels* **2018**, *2*, 1974–1978. https://doi.org/10.1039/C8SE00328A.

(3) Liu, Y.; Ng, S.-M.; Yiu, S.-M.; Lam, W. W. Y.; Wei, X.-G.; Lau, K.-C.; Lau, T.-C. Catalytic Water Oxidation by Ruthenium(II) Quaterpyridine (Qpy) Complexes: Evidence for Ruthenium(III) Qpy-N,N‴-Dioxide as the Real Catalysts . *Angew. Chemie* **2014**, *126* (52), 14696–14699. https://doi.org/10.1002/ange.201408795.

(4) Huber, F. L.; Nauroozi, D.; Mengele, A. K.; Rau, S. Synthesis and Characterization of a Ruthenium(II) Complex for the Development of Supramolecular Photocatalysts Containing Multidentate Coordination Spheres. *Eur. J. Inorg. Chem.* **2017**, 4020–4027. https://doi.org/10.1002/ejic.201700565.

(5) Zhang, G.; Zong, R.; Tseng, H. W.; Thummel, R. P. Ru(II) Complexes of Tetradentate Ligands Related to 2,9-Di(pyrid-2´-yl)-1,10-phenanthroline. *Inorg. Chem.* **2008**, *47* (3), 990–998. https://doi.org/10.1021/ic701798v.

(6) Kowacs, T.; O'Reilly, L.; Pan, Q.; Huijser, A.; Lang, P.; Rau, S.; Browne, W. R.; Pryce, M. T.; Vos, J. G. Subtle Changes to Peripheral Ligands Enable High Turnover Numbers for Photocatalytic Hydrogen Generation with Supramolecular Photocatalysts. *Inorg. Chem.* **2016**, *55* (6), 2685–2690. https://doi.org/10.1021/acs.inorgchem.5b01752.

(7) Neese, F. The ORCA Program System. *Wiley Interdiscip. Rev. Comput. Mol. Sci.* **2012**, *2* (1), 73–78. https://doi.org/10.1002/wcms.81.

(8) Stephens, P. J.; Devlin, F. J.; Chabalowski, C. F.; Frisch, M. J. Ab Initio Calculation of Vibrational Absorption and Circular Dichroism Spectra Using Density Functional Force Fields. *J. Phys. Chem.* **1994**, *98* (45), 11623–11627. https://doi.org/10.1021/j100096a001.

(9) Becke, A. D. Density-Functional Thermochemistry. III. The Role of Exact Exchange. *J. Chem. Phys.* **1993**, *98* (7), 5648–5652. https://doi.org/10.1063/1.464913.

(10) Vosko, S. H.; Wilk, L.; Nusair, M. Accurate Spin-Dependent Electron Liquid Correlation Energies for Local Spin Density Calculations: A Critical Analysis. *Can. J. Phys.* **1980**, *58* (8), 1200–1211. https://doi.org/10.1139/p80-159.

(11) Lee, C.; Yang, W.; Parr, R. G. Development of the Colic-Salvetti Correlation-Energy Formula into a Functional of the Electron Density. *Phys. Rev. B* **1988**, *37* (2), 785–789.

https://doi.org/10.1103/PhysRevB.37.785.

(12) Grimme, S.; Antony, J.; Ehrlich, S.; Krieg, H. A Consistent and Accurate *Ab Initio* Parametrization of Density Functional Dispersion Correction (DFT-D) for the 94 Elements H-Pu. *J. Chem. Phys.* **2010**, *132* (15), 154104. https://doi.org/10.1063/1.3382344.

(13) Grimme, S.; Ehrlich, S.; Goerigk, L. Effect of the Damping Function in Dispersion Corrected Density Functional Theory. *J. Comput. Chem.* **2011**, *32*, 1456–1465. https://doi.org/10.1002/jcc.

(14) Barone, V.; Cossi, M. Quantum Calculation of Molecular Energies and Energy Gradients in Solution by a Conductor Solvent Model. *J. Phys. Chem. A* **1998**, *102* (11), 1995–2001. https://doi.org/10.1021/jp9716997.

(15) Neese, F.; Wennmohs, F.; Hansen, A.; Becker, U. Efficient, Approximate and Parallel Hartree-Fock and Hybrid DFT Calculations. A "Chain-of-Spheres" Algorithm for the Hartree-Fock Exchange. *Chem. Phys.* **2009**, *356* (1–3), 98–109. https://doi.org/10.1016/j.chemphys.2008.10.036.

(16) Izsák, R.; Neese, F. An Overlap Fitted Chain of Spheres Exchange Method. *J. Chem. Phys.* **2011**, *135* (14), 144105. https://doi.org/10.1063/1.3646921.

(17) Neese, F. An Improvement of the Resolution of the Identity Approximation for the Formation of the Coulomb Matrix. *J. Comput. Chem.* **2003**, *24* (14), 1740–1747. https://doi.org/10.1002/jcc.10318.

(18) Petrenko, T.; Kossmann, S.; Neese, F. Efficient Time-Dependent Density Functional Theory Approximations for Hybrid Density Functionals: Analytical Gradients and Parallelization. *J. Chem. Phys.* **2011**, *134* (5), 054116. https://doi.org/10.1063/1.3533441.

(19) Weigend, F. Accurate Coulomb-Fitting Basis Sets for H to Rn. *Phys. Chem. Chem. Phys.* **2006**, *8* (9), 1057–1065. https://doi.org/10.1039/b515623h.

(20) Pantazis, D. A.; Neese, F. All-Electron Basis Sets for Heavy Elements. *Wiley Interdiscip. Rev. Comput. Mol. Sci.* **2014**, *4* (4), 363–374. https://doi.org/10.1002/wcms.1177.

(21) Jäger, M.; Freitag, L.; González, L. Using Computational Chemistry to Design Ru Photosensitizers with Directional Charge Transfer. *Coord. Chem. Rev.* **2015**, *304–305*, 146–165. https://doi.org/10.1016/j.ccr.2015.03.019.

(22) Sánchez-Murcia, P. A.; Nogueira, J. J.; González, L. Exciton Localization on Ru-Based Photosensitizers Induced by Binding to Lipid Membranes. *J. Phys. Chem. Lett.* **2018**, *9* (4), 683–688. https://doi.org/10.1021/acs.jpclett.7b03357.

(23) Kupfer, S.; Guthmuller, J.; Wächtler, M.; Losse, S.; Rau, S.; Dietzek, B.; Popp, J.; González, L. Protonation Effects on the Resonance Raman Properties of a Novel (Terpyridine)Ru(4H-Imidazole) Complex: An Experimental and Theoretical Case Study. *Phys. Chem. Chem. Phys.* **2011**, *13* (34), 15580–15588. https://doi.org/10.1039/c1cp21521c.

(24) Vlček, A.; Záliš, S. Modeling of Charge-Transfer Transitions and Excited States in d^6 Transition Metal Complexes by DFT Techniques. *Coord. Chem. Rev.* **2007**, *251* (3–4), 258–287. https://doi.org/10.1016/j.ccr.2006.05.021.

(25) Batlogg, A.; Fumanal, M. Computational Assessment of MLCT versus MC Stabilities in First-to-Third-Row d^6 Pseudo-Octahedral Transition Metal Complexes. *J. Comput. Chem.* **2019**, *40* (27), 2377–2390. https://doi.org/10.1002/jcc.26014.

(26) Coe, B. J.; Pilkington, R. A. Theoretical Studies on Two-Dimensional Nonlinear Optical Chromophores with Pyrazinyl Cores and Organic or Ruthenium(II) Ammine Electron Donors. *J. Phys. Chem. A* **2014**, *118* (12), 2253–2268. https://doi.org/10.1021/jp4114927.

(27) Weigend, F.; Ahlrichs, R. Balanced Basis Sets of Split Valence, Triple Zeta Valence and Quadruple Zeta Valence Quality for H to Rn: Design and Assessment of Accuracy. *Phys. Chem. Chem. Phys.* **2005**, *7* (18), 3297–3305. https://doi.org/10.1039/b508541a.

(28) Andrae, D.; Häußermann, U.; Dolg, M.; Stoll, H.; Preuß, H. Energy-Adjusted Ab Initio Pseudopotentials for the Second and Third Row Transition Elements. *Theor. Chim. Acta* **1990**, *77* (2), 123–141. https://doi.org/10.1007/BF01114537.

(29) Garcia-Ratés, M.; Neese, F. Effect of the Solute Cavity on the Solvation Energy and Its Derivatives within the Framework of the Gaussian Charge Scheme. *J. Comput. Chem.* **2020**, *41* (9), 922–939. https://doi.org/10.1002/jcc.26139.

(30) Hirata, S.; Head-Gordon, M. Time-Dependent Density Functional Theory within the Tamm-Dancoff Approximation. *Chem. Phys. Lett.* **1999**, *314* (3–4), 291–299. https://doi.org/10.1016/S0009-2614(99)01149-5.

(31) van Wüllen, C. Molecular Density Functional Calculations in the Regular Relativistic Approximation: Method, Application to Coinage Metal Diatomics, Hydrides, Fluorides and Chlorides, and Comparison with First-Order Relativistic Calculations. *J. Chem. Phys.* **1998**, *109* (2), 392–399. https://doi.org/10.1063/1.476576.

(32) Pantazis, D. A.; Chen, X. Y.; Landis, C. R.; Neese, F. All-Electron Scalar Relativistic Basis Sets for Third-Row Transition Metal Atoms. *J. Chem. Theory Comput.* **2008**, *4* (6), 908–919. https://doi.org/10.1021/ct800047t.

(33) Mai, S.; Richter, M.; Heindl, M.; Menger, M. F. S. J.; Atkins, A.; Ruckenbauer, M.; Ibele, L. M.; Kropf, S.; Oppel, M.; Marquetand, L.; González, L. SHARC2.1: Surface Hopping Including Arbitrary Couplings – Program Package for Non-Adiabatic Dynamics. sharc-md.org (2019).

(34) Dahl, J. P.; Springborg, M. The Morse Oscillator in Position Space, Momentum Space, and Phase Space. *J. Chem. Phys.* **1988**, *88* (7), 4535–4547. https://doi.org/10.1063/1.453761.

(35) Zobel, J. P.; Nogueira, J. J.; González, L. Finite-Temperature Wigner Phase-Space Sampling and Temperature Effects on the Excited-State Dynamics of 2-Nitronaphthalene. *Phys. Chem. Chem. Phys.* **2019**, *21* (26), 13906–13915. https://doi.org/10.1039/c8cp03273d.

(36) Plasser, F.; Wormit, M.; Dreuw, A. New Tools for the Systematic Analysis and Visualization of Electronic Excitations . I . Formalism *J. Phys. Chem.* **2014**, *141*, 024106. https://doi.org/10.1063/1.4885819.

(37) Plasser, F. TheoDORE: A package for theoretical density, orbital relaxation, and exciton analysis http://theodore-qc.sourceforge.net (accessed Jun 9, 2020).

(38) Plasser, F. TheoDORE: A Toolbox for a Detailed and Automated Analysis of Electronic Excited State Computations. *J. Chem. Phys.* **2020**, *152* (8), 084108. https://doi.org/10.1063/1.5143076.

(39) Mai, S.; Plasser, F.; Dorn, J.; Fumanal, M.; Daniel, C.; González, L. Quantitative Wave Function Analysis for Excited States of Transition Metal Complexes. *Coord. Chem. Rev.* **2018**, *361*, 74–97. https://doi.org/10.1016/j.ccr.2018.01.019.

(40) Zong, R.; Thummel, R. P. 2,9-Di-(2′-pyridyl)-1,10-phenanthroline: A Tetradentate Ligand for Ru(II). *J. Am. Chem. Soc.* **2004**, *126*, 10800–10801. https://doi.org/10.1021/ja047410y.
